# The CD64/CD28/CD3ζ chimeric receptor reprograms T-cell metabolism and promotes T-cell persistence and immune functions while triggering antibody-independent and antibody-dependent cytotoxicity

**DOI:** 10.1186/s40164-025-00601-2

**Published:** 2025-02-17

**Authors:** Sara Caratelli, Francesca De Paolis, Domenico Alessandro Silvestris, Silvia Baldari, Illari Salvatori, Apollonia Tullo, Giulia Lanzilli, Aymone Gurtner, Alberto Ferri, Cristiana Valle, Simona Padovani, Valeriana Cesarini, Tommaso Sconocchia, Loredana Cifaldi, Roberto Arriga, Giulio Cesare Spagnoli, Soldano Ferrone, Adriano Venditti, Piero Rossi, Graziano Pesole, Gabriele Toietta, Giuseppe Sconocchia

**Affiliations:** 1 Department of Biomedicine, Institute of Translational Pharmacology, Italian National Research Council (CNR), Via Fosso del Cavaliere 100, Rome, 00133 Italy; 2 Dipartimento di Bioscienze, Biotecnologie e Ambiente, University of Bari, Bari, Italy; 3 Tumor Immunology and Immunotherapy Unit, IRCCS Regina Elena National Cancer Institute, Rome, Italy; 4 IRCCS Fondazione Santa Lucia, Rome, Italy; 5 Department of Biomedicine, Institute of Biomembranes Bioenergetics and Molecular Biotechnologies, National Research Council (CNR), Bari, Italy; 6 Saint Camillus, International Medical University (UNICAMILLUS), Rome, Italy; 7 San Raffaele Telethon Institute for Gene Therapy (SR-TIGET), IRCCS San Raffaele Scientific Institute, Milan, Italy; 8 Department of Clinical Sciences and Translational Medicine, University of Rome “Tor Vergata”, Rome, Italy; 9 Department of Systems Medicine, University of Rome “Tor Vergata”, Rome, Italy; 10 Department of Surgery, Massachusetts General Hospital, Harvard Medical School, Boston, NA USA; 11 Department of Biomedicine and Prevention, University of Rome “Tor Vergata”, Rome, Italy; 12 Department of Experimental Medicine and Surgery, University of Rome “Tor Vergata”, Rome, Italy

**Keywords:** CD64, CAR-T cell, Ligand, Biosensor, Cancer, Cytotoxicity, PD-L1, IFNγ, HLA-DR, Lactate, LDH, Glycolysis

## Abstract

**Background:**

Recent studies have shown that CD32/CD8a/CD28/CD3ζ chimeric receptor cells directly kill breast cancer cells, suggesting the existence of cell surface myeloid FcγR alternative ligands (ALs). Here, we investigated the metabolism, ALs, cytotoxicity, and immunoregulatory functions of CD64/CD28/CD3ζ in colorectal cancer (CRC) and squamous cell carcinoma of the head and neck.

**Methods:**

The CD64/CD28/CD3ζ -SFG retroviral vector was used to produce viruses for T-cell transduction. T-cell expansion and differentiation were monitored via flow cytometry. Gene expression was assessed by RNA-seq. Bioenergetics were documented on a Seahorse extracellular flux analyzer. CD64/CD28/CD3ζ polarization was identified via confocal microscopy. Cytotoxicity was determined by MTT assay and bioluminescent imaging, and flow cytometry. Tridimensional antitumor activity of CD64/CD28/CD3ζ T cells was achieved by utilizing HCT116-GFP 3D spheroids via the IncuCyte S3 Live-Cell Analysis system. The intraperitoneal distribution and antitumor activity of NIR-CD64/CD28/CD3ζ and NIR-nontransduced T cells were investigated in CB17-SCID mice bearing subcutaneous FaDu Luc + cells by bioluminescent and fluorescent imaging. IFNγ was assessed by ELISA.

**Results:**

Compared to CD16/CD8a/CD28/CD3ζ T cells, CD32/CD8a/CD28/CD3ζ T cells, and non-transduced T cells, CD64/CD28/CD3ζ T cells exhibited the highest levels of cell expansion and persistence capacity. A total of 235 genes linked to cell division and 52 genes related to glycolysis were overexpressed. The glycolytic phenotype was confirmed by functional in vitro studies accompanied by preferential T-cell effector memory differentiation. Interestingly, oxamic acid was found to inhibit CD64-CR T cell proliferation, indicating the involvement of lactate. Upon CD64/CD28/CD3ζ T-cell conjugation with CRC cells, CD64/CD28/CD3ζ cells polarize at immunological synapses, leading to CRC cell death. CD64/CD28/CD3ζ T cells kill SCCHN cells, and in combination with the anti-B7-H3 mAb (376.96) or anti-EGFR mAb, these cells trigger antibody-dependent cellular cytotoxicity (ADCC) in vitro under 2D and 3D conditions. The 376.96 mAb combined with CD64/CD28/CD3ζ T cells had anti-SCCHN activity in vivo. In addition, they induce the upregulation of PD-L1 and HLA-DR expression in cancer cells via IFNγ. PD-L1 positive SCCHN cells in combination with anti-PD-L1 mAb and CD64-CR T cells were killed by ADCC, which enhanced direct cytotoxicity. These findings indicate that the glycolytic phenotype is involved in CD64-CR T cell proliferation/expansion. These cells mediate long-lasting HLA-independent cytotoxicity and ADCC in CRC and SCCHN cells.

**Conclusions:**

CD64/CD28/CD3ζ T cells could significantly impact the rational design of personalized studies to treat CRC and SCCHN and the identification of novel FcγR ALs in cancer and healthy cells.

**Supplementary Information:**

The online version contains supplementary material available at 10.1186/s40164-025-00601-2.

## Introduction

The impressive success of chimeric antigen receptor (CAR) T-cell-based immunotherapy in B-cell malignancies [1] has sparked extraordinary interest in the scientific and medical communities [2–5]. In contrast, CAR-T-cell-based immunotherapy for solid tumors has not been successful due to cellular, metabolic, and anatomical complexities and tissue heterogeneity, which contribute to shaping the hostile tumor microenvironment (TME) for resident immune cells [6–9]. Despite their doubtless antitumor activity, CAR-T cells have also shown some limitations. In addition to toxicity and high costs, manufacturing problems hinder the use of these materials in therapy [10]. For instance, some subgroups of patient CAR-T-cell products failed to generate satisfactory cell expansion in vitro and in vivo and/or showed limited persistence “in vivo”, often involving patients previously subjected to multiple chemotherapies. CAR-T-cell persistence appears to be regulated by the type of costimulatory molecule utilized for the generation of CAR constructs [11–14]. Additionally, metabolic reprogramming of cancer cells into hypoxic and aerobic glycolytic phenotypes results in high lactate levels, which induces TME acidification, leading to tumor growth, metastasis, and inhibition of T-cell functions (proliferation, activation, differentiation, and apoptosis) [15, 16]. However, evidence has also been reported that an aerobic glycolytic phenotype can enhance T-cell differentiation and the functions of CD8 + T cells [17]. To date, antigen loss or downregulation is a common limitation of tumor resistance in CAR-T cells [18]. Thus, the development of CAR-T cells capable of efficient proliferation, resistance to the glycolytic TME, specificity, and optimal cytotoxic functions may help investigators overcome these limitations.

One interesting experimental strategy aimed at redirecting T cells against target cells involves engineering T cells with the CD16/gamma receptor gene, which can eliminate cancer cells in combination with an anti-TAA monoclonal antibody (mAb) produced by the ADCC [19]. Thus, we and others have generated CD16-chimeric receptors (CRs) composed of extracellular CD16 linked to a transmembrane CD8α fused to the T-cell costimulatory molecule CD28/CD3ζ chain. In combination with therapeutic mAbs, CD16-CR T cells were successfully redirected toward hematologic and solid cancer cells [20–23]. Recently, T cells engineered with CD16 valine/CD28/CD3ζ, in combination with the anti-CD20 mAb rituximab were tested in a phase I clinical study of CD20-positive non-Hodgkin lymphoma. Its administration appears to be safe, as it is associated with limited cytokine release syndrome (CRS) and neurotoxicity [24]. In addition to ADCC, FcγRs trigger a variety of other biological functions, including phagocytosis [25], tissue remodeling induced by ischemic and thermic damage, and inflammation following cytokine production [26–28]. The type and extent of these functions are regulated by a variety of factors, including the affinity of the antibody for the FcγR expressed on effector cells [29]. The FcγR family includes several types of FcγRs. CD16 and CD32 are low-affinity receptors characterized by two extracellular Ig-like domains. CD16 is expressed mainly on NK cells and to a lesser extent on a monocyte subset, while CD16B is expressed on granulocytes. The low-affinity receptors CD32A and CD32C (activatory) are typically expressed on myeloid monocytic cells, while CD32B (inhibitory) is expressed on B cells. Both IgG-containing complexes and IgGs that are bound to the cell surface bind to these complexes [30]. CD16 and CD32 are polymorphic. The best-known polymorphisms are CD16^158F^, CD16^158V^, CD32^131R^, and CD32^131H^, of which CD16^158V^ and CD32^131R^ have the highest affinity for the Fc fragment of the Abs. Interestingly, CD32A and CD32C are the only monomeric FcγRs that can directly transduce intracellular signals through the immunoreceptor tyrosine-based activation motif (ITAM) and easily bind to the IgG2a opsonized epidermal growth factor receptor (EGFR), leading to antibody-dependent cellular cytotoxicity (ADCC) [30, 31].

CD64 is a unique FcγR with a high affinity for monomeric IgGs, and unlike the other members, it is composed of three extracellular Ig-like domains (D1, D2, and D3). CD64 is constitutively expressed on monocytes/macrophages and dendritic cells (DCs), while neutrophils and mast cells express CD64 after stimulation with interferon-gamma (IFNγ) or granulocyte colony-stimulating factor (G-CSF) [32–34]. FcγR negative target cells coated with mAbs, in the presence of CD64 positive effector cells, result in the activation of the ADCC. CD64 binds IgG1, IgG3, and IgG4 but has no affinity for IgG2 [35]. Thus, to further improve Fcγ-CR technology, we produced a comprehensive set of Fcγ-CRs capable of binding with different affinities, the majority of which are currently available therapeutic mAbs. This set of Fcγ−CRs includes the standard CD16-CR, polymorphic CD16-CR, standard extracellular CD32-CR, polymorphic CD32-CR, and extracellular CD64-CR, hereafter referred to as CD64-CR. All of them possess an intracellular CD28/CD3ζ, and each Fcγ-CR can be utilized in combination with the appropriate therapeutic mAb based on the antigen specificity and the binding affinity of the Fc fragment. For instance, cetuximab (IgG1) can kill EGFR + cells either directly or indirectly by redirecting NK cells against cancer cells through CD16A. However, panitumumab (IgG2a) can kill EGFR + cells only directly since CD16A on NK cells does not bind to IgG2a. Therefore, T cells engineered with a CD32-CR can overcome such limitations by allowing the use of panitumumab when needed [20, 21]. To date, FcγRs are known to bind the Ig Fc fragment and pentraxins [36, 37]. The FcγR/pentraxin interaction promotes leukocyte-mediated phagocytosis and cytokine production [38, 39]. However, previous studies have shown that CD16A mediates antibody-independent cytotoxicity by NK cells, suggesting the presence of alternative ligands (ALs) of CD16A on the surface of cancer cells [40]. In contrast, it is still unclear whether the typical myeloid monocytes expressing CD32 and CD64 can directly recognize the cell surface ligands of malignant cells. Indeed, utilizing CD32-CR as a biosensor, we previously demonstrated, for the first time, that CD32-CR T cells specifically recognized ALs expressed on the surface of epithelial cancer cells. CD32-CR T cells preferentially kill sensitive breast cancer (BC) cells, leading to their elimination in vitro and *in vivo.* CD32-CR*-*T cells at least mediate granule-dependent cytotoxicity, but this effect requires ICAM1 expression on target cells.

These data provide evidence of the existence of myeloid FcγR surface ligands in cancer cells [41]. Based on this background, we asked whether CD64 ALs are also expressed by cancer cells. To this end, we utilized CD64-CR T cells as biosensors. Here, we show, for the first time, that such recognition takes place in the unexpected context of a specific enhancement of the cell expansion which was linked to glycolytic reprogramming of CD64-CR T cell and involved lactate. These cells displayed a prevalent T effector memory (Tem) differentiation profile and direct elimination of CRC and SCCHN cells in the absence of Abs. Moreover, CD64-CR T cells, in combination with an anti-B7-H3 mAb or anti-EGFR mAb, exhibited antitumor activity in vivo. In addition, CD64-CR T cells exhibit strong immunoregulatory functions since they induce the upregulation of programmed death-ligand 1 (PD-L1) and *de novo* expression of HLA-DR on cancer cells through the specific production of IFNγ. Remarkably, CD64-CR-T-cell cytotoxicity was enhanced in vivo and in vitro when combined with the anti-B7-H3 mAb and in vitro when combined with the anti-PD-L1 mAb.

While further studies are needed to determine the nature of CD64-CR ALs in cancer and healthy cells, our data suggest that these cells may be useful as a platform for designing innovative, highly effective immunotherapies for CRC and SCCHN.

## Materials and methods

### Antibodies and reagents

Allophycocyanin (APC)-conjugated mouse anti-human CCR7 Clone 2L1A (566762), fluorescein-5-isothiocyanate (FITC)-conjugated mouse anti-human CD3 Clone UCHT1 (555332), phycoerythrin (PE)-conjugated mouse anti-human CD64 Clone 10.1 (558592), PerCP-Cy™5.5-conjugated mouse anti-human CD4 Clone SK3 (332772), APC-conjugated mouse anti-human CD8 Clone RPA-T8 (555369), Brilliant Violet (BV) 605-conjugated mouse anti-human CD4 (562658), BV786-conjugated mouse anti-human CD8 (563823), PerCP-Cy™5.5-conjugated mouse anti-human CD45RA Clone HI100 (563429), BV711-conjugated mouse anti-human CD45RO (563722), FITC-conjugated mouse anti-human CD62L Clone DREG-56 (555543), V450-conjugated mouse anti-human CD95 (561632), V500-conjugated mouse anti-human CD27 (561222), PE-conjugated mouse anti-human PD-L1 (557924), PE-conjugated mouse anti-human HLA-DR (555812), FITC-conjugated Annexin V (556420), propidium iodide (PI) staining solution (51-66211E), purified NA/LE mouse anti-human CD3 Clone UCHT1 (555329) and purified NA/LE mouse anti-human CD28 Clone CD28.2 (555725) were purchased from BD Bioscience (San Jose, CA, USA). The anti-human B7-H3 (CD276) mAb clone 376.96 was developed and characterized as previously described [42]. The anti-EGFR mAbs cetuximab (Erbitux) and panitumumab (Vectibix) were obtained from Merck KGaA (Darmstadt, Germany) and Amgen (Thousand Oaks, CA, USA), respectively. Atezolizumab (Tecentriq) was obtained from Roche (Basel Switzerland). The goat anti-human IFNGR1 polyclonal antibody (PA5-47866) was obtained from Thermo Fisher Scientific (Waltham, MA, USA). Human recombinant interleukin-7 (IL-7) (130-095-367), human recombinant interleukin-15 (IL-15) (130-095-760), and human CD56 microbeads (130-050-401) were purchased from Miltenyi Biotec (Bergisch Gladbach, Germany). For DAPI staining, fluoromount aqueous mounting medium and 3-(4,5-dimethylthiazol-2-Yl)-2,5-diphenyltetrazolium bromide (MTT) were obtained from Merck KGaA (Darmstadt, Germany). Retro-Nectin (a recombinant human fibronectin) was purchased from Takara Bio (Kusaka, Japan). D-luciferin and near-infrared (NIR) fluorescent cell labeling dye (XenoLight DiR, cat. 125964) were obtained from PerkinElmer (Waltham, MA, USA). Oxamic Acid was obtained from MedChemExpress (NJ USA). Roswell Park Memorial Institute (RPMI) 1640, McCoy’s 5a Medium Modified (McCoy’s 5 A), Dulbecco’s Modified Eagle’s Medium (DMEM), Eagle’s Minimum Essential Medium (EMEM) cell culture media, fetal bovine serum (FBS), L-glutamine, and penicillin/streptomycin were obtained from Euroclone (Pero, MI, Italy).

### Cell lines

The human CRC cell lines HCT116 (RRID: CVCL_0291) and HT-29 (RRID: CVCL_0320) were cultured in RPMI medium and DMEM, respectively. The hypopharyngeal squamous cell carcinoma FaDu cell line (RRID: CVCL_1218) was maintained in EMEM, and submandibular gland squamous cell carcinoma A-253 cells (RRID: CVCL_1060) were cultured in McCoy’s 5 A. All the above-mentioned media were supplemented with 10% FBS, 2 mM L-glutamine, 0.1 mg/ml streptomycin, and 100 U/ml penicillin to obtain complete cell culture media (CM). The CRC cell lines HCT116 and HT-29 were obtained from our laboratory. HCT116 Luc + cells were purchased from Caliper Life Sciences (Caliper Life Sciences, Inc., Hopkinton, MA, USA). HCT116 GFP cells were obtained from the parental cell line transduced with pLenti 62 emGreen, followed by blasticidin (5 μg/ml) selection. Cell lines were passaged twice a week and cultured for a maximum of 6–8 weeks. All cancer cell lines were tested negative for mycoplasma and authenticated by PCR-locus-technology in 2024 (Eurofins, Ebersberg, Germany).

### Chimeric receptors

The CD64-CR construct was synthesized by the GeneArt Gene Synthesis Service (Thermo Fisher Scientific, Waltham, MA, USA). The chimera was generated by joining the extracellular region of CD64 with CD28 and the endodomain of CD3ζ. The leader sequence from mouse CD64 was included in the 5’ end of the CD64 extracellular region. After synthesis, the gene cassette was cloned and inserted into the NcoI and MluI sites of the SFG retroviral vector. The CD16-CR and CD32-CR constructs were constructed as previously described [20, 21].

### Retrovirus production and human T-cell transduction

Retroviral supernatant production was performed as previously described [20, 21]. Peripheral blood mononuclear cells (PBMCs) were isolated from our PBMC cell bank, and NK cells were removed utilizing human CD56 microbeads. T cells were cultured for 72 h in non-tissue culture-treated 24-well plates precoated with 1 μg/ml of anti-human CD3 antibody and 1 μg/ml of anti-human CD28 antibody. Activated T cells were seeded into a retroviral-preloaded 24-well plate for 72 h at 37 °C in 5% CO_2_. After transduction, the T cells were expanded in RPMI-1640 complete medium supplemented with 10 ng/ml IL-7 and 5 ng/ml IL-15 for further analysis.

### RNA sequencing of CD64-CR T cells and non-transduced (NT) T cells

RNA-seq libraries were prepared from 300 ng of total RNA using the Illumina^®^ Stranded Total RNA Prep Ligation Kit with Ribo-Zero Plus Preparation Kit (Illumina, San Diego, CA, USA) according to the manufacturer’s protocol. The first step of the protocol consists of the removal of ribosomal RNA using the Ribozero plus: the DNA probes capture the ribosomal RNA, forming a double strand that can be degraded by RNaseH, and the DNA probes are eliminated enzymatically. In the second step, RNA is fragmented and denatured. The first and second strands of cDNA were synthesized and then subjected to three steps consisting of 3’ end adenylation, anchor ligation, and library amplification. The cDNA libraries were checked on a Bioanalyzer 2100 and quantified by fluorimetry using a Qubit dsDNA High Sensitivity Kit (Thermo Fisher Scientific) on a Qubit Fluorometer (Thermo Fisher Scientific). Sequencing was performed on the NovaSeq 6000 platform, generating approximately 100 million of 150 bp paired-end reads for each sample.

### Bioinformatics analysis

In the preprocessing step, the raw reads in FASTQ format were inspected and cleaned using FASTP [43]. The mean quality per base cutoff was fixed at a phred score of 20, and reads with more than 30% unqualified bases (-q 20 -u 30 -l 55 –detect_adapter_for_pe) were removed. Reads shorter than 55 bases were also removed. Cleaned reads were aligned with STAR (2.7.9a) [44] using the ENCODE standard options onto a consensus version of the reference human genome (GRCh38.p14 primary assembly PAR masked). The 1000 Genomes Project variant call format file with consensus single-nucleotide variants and insertion/deletions (InDels) was generated at the genome generation stage, and the alternative alleles in this variant call format were inserted into the reference genome to create a “transformed” genome. At the mapping stage, the reads were mapped to the transformed genome, and the alignments were subsequently transformed back to the original coordinates. Gene expression was quantified with featureCounts [45], summarizing the exonic reads according to the Gencode comprehensive (release 44) gene annotation taking advantage of the strand-oriented nature of the reads. The row counts were normalized with DESeq2 [46](1.42.0), and differential gene expression was tested with “time” as a covariate. The *degPatterns* function of the R library DEGreport was used to identify clusters of genes with similar gene expression trends across the three times considered. Heatmaps were generated with the R Pheatmap library, which also highlighted clusters of coregulated genes. Gene set enrichment was tested with the R library clusterProfiler [47]. Pathview [48] was used for pathway-based data integration and visualization.

### Flow cytometry

Phenotypic analysis of CD64-CR-engineered T cells was performed by flow cytometry. After transduction, the T cells were incubated for 30 min at 4 °C with mAbs specific for CD8, CD4, CD64, CD45RA, CD45RO, CD62L, CCR7, CD27 and CD95 conjugated with BDHBV786, BDHBV605, PE, PerCP-Cy5.5, BDHBV711, FITC, APC, BDHV500, and BDHV450 fluorochromes, respectively (see above).

To perform a flow cytometry cytotoxicity assay, HCT116 cells were incubated with CD64-CR-transduced or nontransduced (NT) T cells at an E: T ratio of 2:1 with or without 1 μg/ml cetuximab or the 376.96 mAb. After 18 h, the cells were collected and stained with APC-conjugated mouse anti-human CD3, FITC-conjugated Annexin V, and PI solution for 15 min at room temperature in the dark. The percentage of apoptotic target cells was determined by posting an electronic gate on CD3- Annexin V + and PI + cells.

Samples were acquired with a BD FACSCalibur™, BD FACSLyric (Becton Dickinson, Franklin Lakes, NJ, USA), and Beckman Coulter Cytoflex flow cytometer. All the data were analyzed using Tree Star, Inc., FlowJo software.

### Bioenergetic analysis of CD64-CR T cells and NT T cells

Bioenergetic analyses of CD64-CR T and NT T cells were conducted using a Seahorse XF96e Analyzer (Seahorse Bioscience Agilent, Santa Clara, CA, USA) through a real-time ATP assay, cell mito stress test, and glycolysis stress test. All assays were performed according to Agilent’s recommendations. CD64-CR T and NT T cells were seeded (0.5 × 10^6 cells/well) in a 96-well Seahorse plate for all assays.

Briefly, for the Cell Mito Stress Test and ATP Real-Time Rate Assay, the growth medium was replaced with Seahorse XF DMEM (Seahorse Bioscience—Agilent, Santa Clara, CA, USA) supplemented with 1 mM pyruvate, 10 mM glucose, and 2 mM L-glutamine, with the pH adjusted to 7.4. For the Glycolysis Stress Test, the growth medium was replaced with XF test medium (Eagle’s modified Dulbecco’s medium; Agilent Seahorse, Santa Clara, CA, USA) supplemented with 2 mM L-glutamine, and the pH was adjusted to 7.4. Before the assays, the cells were incubated in a 37 °C incubator without CO2 for 45 min to allow them to preequilibrate with the assay medium.

The ATP Real-Time Rate Assay was initiated by measuring the baseline oxygen consumption rate (OCR) and extracellular acidification rate (ECAR). Sequential measurements of the OCR and ECAR were then performed following the addition of 1.5 μM oligomycin, followed by 0.5 μM oligomycin and 0.5 μM antimycin A. The Cell Mito Stress Test began with baseline OCR measurements, followed by sequential OCR assessments after the injection of 1.5 μM oligomycin, 1 μM FCCP (carbonyl cyanide 4-(trifluoromethoxy) phenylhydrazone), and a combination of 0.5 μM rotenone and 0.5 μM antimycin A. For the Glycolysis Stress Test, ECAR measurements were conducted after the addition of 10 mM glucose, 1 μM oligomycin A, and 50 mM 2-deoxyglucose (2-DG).

All the data were analyzed using XFe Wave software 2.6 and are presented as point-to-point ECAR and OCR values normalized to the number of cells. Statistical analysis was performed using Prism software (GraphPad Prism 6 software).

### Immunofluorescence and confocal microscopy

CD64-CR T cells were incubated with HCT116 or HT-29 cells (E: T 2:1) for 1 h, fixed for 10 min with 3% (w/v) formaldehyde in PBS, permeabilized for 10 min with 0.15% Triton, blocked for 1 h in 5% bovine serum albumin, and immunostained with a mouse anti-human CD64 mAb (1:40, BD 555525) overnight at 4 °C in 1% bovine serum albumin and with a Cy3-conjugated donkey anti-mouse antibody at room temperature for 1 h (Jackson ImmunoResearch Laboratories). DNA was counterstained with 0.4 mg/ml DAPI. Images were recorded by using a Zeiss LSM 880 confocal laser scanning microscope equipped with a 60X/1.23 NA oil immersion objective.

### MTT assay

The target cells were cocultured in 96-well plates with CD64 CR or NT T cells at different effector: target (E: T) ratios in the presence or absence of 1 μg/ml cetuximab, panitumumab, the anti-human B7-H3 (376.96) mAb, or 100 ng/ml atezolizumab for 72 h at 37 °C in triplicate. After incubation, the non-adherent effector cells were removed, and the tumor target cell viability was determined via the MTT assay as previously described [20, 21, 41].

### 3D tumor spheroid generation and analysis

HCT116 GFP cells, at a density of 1 × 10^3^ cells/well, were seeded for 72 h in 96-well ultralow adherence plates (Corning, #7007) to allow 3D spheroid formation, with a single spheroid settling in the center of each well. Then, CD64-CR T or NT T cells were added to HCT116 GFP 3D spheroids at different E: T ratios (10:1; 5:1; 2.5:1; 1.25:1) in the presence or absence of 1 μg/ml cetuximab or an anti-human B7-H3 (376.96) mAb. The cells were cultured using the IncuCyte S3 Live-Cell Analysis System (Sartorius, Essen Bioscience) for real-time imaging of spheroids in triplicate. Images were collected, and the intensity of GFP fluorescence was analyzed using IncuCyte 2022B Rev2 software.

### In vitro bioluminescence imaging (BLI)

HCT116 Luc + cells were seeded in 96-well microplates in triplicate and incubated with CD64-CR or NT T cells at different E: T ratios. After 3 days of coculture, the cell culture medium was supplemented with D-luciferin (150 μg/mL) for 10 min, and the analysis was performed using the IVIS^®^ Lumina II platform (PerkinElmer, Waltham, MA, USA). Photons emitted from Luc + cells in selected regions of interest (ROIs) were quantified using Living Image^®^ software.

### Xenograft mouse model

Rabbit anti-asialo-GM1 antibody deplete NK cells in vivo (WAKO Chemical Europe, Neuss Germany). Endogenous NK cell activity was suppressed by intraperitoneal injection of a 20 μl rabbit anti-asialo-GM1 antibody. Mice received anti-asialo-GM1 antibody on days − 3, 0, + 14, and + 21 since tumor cell engraftment [49].

FaDu cells were transduced with a third-generation self-inactivating lentiviral vector expressing firefly luciferase followed by blasticidin (5 μg/ml) selection (FaDu Luc^+^) [50]. To perform in vivo fluorescent tracking of T cells, both NT- and CD64-CR-transduced T cells were labeled with near-infrared (NIR) fluorescent cell labeling dye (XenoLight DiR) according to the manufacturer’s instructions.

Eight-week-old immunodeficient SCID (CB17/Icr-Prkdc^scid^/IcrIcoCrl) female mice were purchased from Charles River Laboratories (Calco, Italy). Animals were fed a low-fluorescence purified diet based on the AIN-93G formulation (Mucedola srl, Settimo Milanese, Italy) for approximately 2 weeks before experimentation involving fluorescence imaging. FaDu Luc^+^ cells (5.0 × 10^5^) were implanted in the dorsal subcutaneous area, and tumor growth was monitored via bioluminescence 1 week later. After 10 days of tumor growth, the animals were randomly divided into 5 groups (*n* = 3/group). Group 1 served as a control and received an equivalent volume (200 μl) of saline solution by intraperitoneal administration (ip); group 2 received 2.5 × 10^6^ NIR (Near-InfraRed cell tracker labeled)-NT T cells ip; group 3 received 2.5 × 10^6^ NIR-CD64-CR transduced T cells ip; group 4 received 150 μg of 376.96 mAb and, after 1 h, 2.5 × 10^6^ NIR-NT T cells; group 5 received 150 μg of 376.96 mAb and, after 1 h, 2.5 × 10^6^ NIR-CD64-CR T cells. Three days before and on the day of T-cell administration, the mice received 20 μl of rabbit anti-asialo-GM1 antibody intraperitoneally. Bioluminescent imaging (BLI) and fluorescent imaging (FLI) were performed using an IVIS^®^ Spectrum system equipped with Living Image^®^ software for data quantification (PerkinElmer) immediately after T-cell injection and then at 24 and 48 h postadministration.

To assess the antitumor activity of CD64-CR T cells, FaDu Luc + cells were inoculated in the dorsal region of CB17-SCID immunodeficient mice as described above. Animals were randomly divided into 6 groups (*n* =4-5/group): 12 (1st dose) and 24 (2nd dose) days after tumor cell injection. The groups were divided into the following groups: group 1, saline; group 2, ip 2.5 × 10^6^ NT T cells; group 3, ip 2.5 × 10^6^ CD64-CR-transduced T cells; group 4, ip 150 μg of 376.96 mAb; group 5, ip 150 μg of 376.96 mAb and after 1 h, 2.5 × 10^6^ NT T cells; and group 6, received ip 150 μg of 376.96 mAb and after 1 h, 2.5 × 10^6^ CD64-CR T cells. Tumor growth was evaluated by BLI before and after the treatments. To be noted, the mice were randomly divided into experimental groups. However, the tumor engraftment and development varied among the animals, even though the administration of cells was performed simultaneously and with the same number of cells for each. To address these differences in tumor progression, we longitudinally monitored each animal individually. We expressed the luminescence at each time point as a ratio compared to the luminescence measured in the same animal at the previous time point. This approach enables us to assess tumor progression on an individual animal basis.

The animal experimental procedures conformed to Italian D. Lgs 26/2014, application of the EU Directive 2010/63/EU and were approved by the IRCCS Regina Elena National Cancer Institute Animal Care and Use Committee (Authorization no. 1109/2020-PR, November 2020).

### ELISA

CD64-CR and NT T cells were incubated with tumor target cells for 24 h at an E: T ratio of 1:1. Then, the supernatants were collected and stored at -20 °C. After thawing, the amount of IFNγ was quantified using a human IFNγ ELISA kit (cat. KHC4021, Invitrogen) following the manufacturer’s instructions.

### Statistical analysis

The data sets were analyzed using GraphPad Prism Software. Unpaired two-tailed t tests and one-way or two-way ANOVA were used to assess statistical significance. Differences with a *p* value < 0.05 were considered to indicate statistical significance.

## Results

### CD64-CR induces persistent T-cell proliferation and expansion ex vivo

**Fig. 1:**
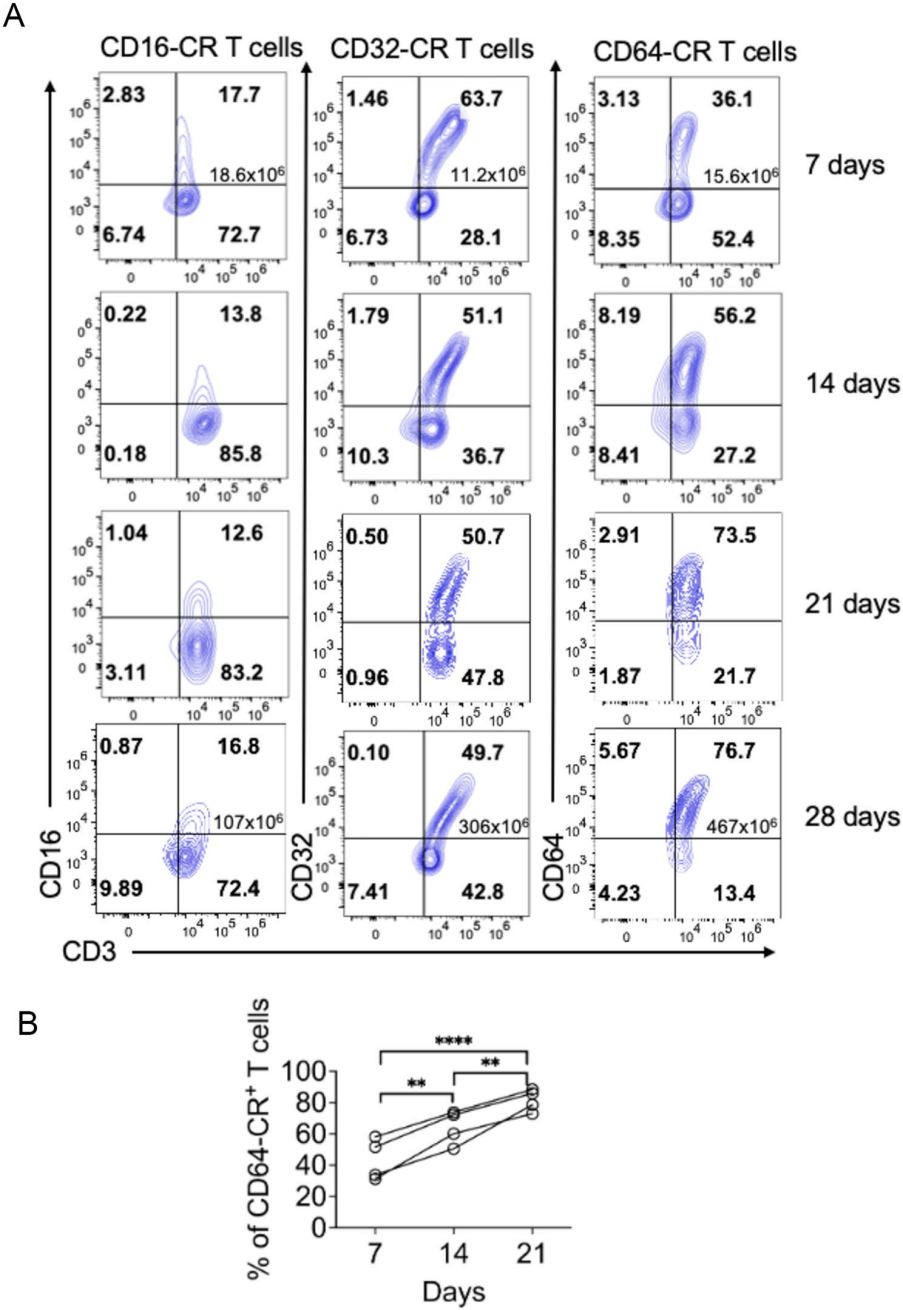
The CD64-CR is the major driver of the proliferation, expansion, and persistence of Fcγ-CR/CD28/CD3ζ T cells. (**A**) CD3-activated human T cells were transduced with CD16-CR, CD32-CR, or CD64-CR. The cell surface expression of Fcγ-CRs was evaluated over time utilizing specific PE-conjugated mouse anti-human FcγRs and FITC-conjugated mouse anti-human CD3 antibodies. The cells were subsequently analyzed via flow cytometry. The numbers in the quadrants show the percentage of cells, whereas the numbers in the upper right quadrants show the total number of cells counted at 7 and 28 days of culture in vitro, regardless of the expression of the indicated markers. (**B**) In vitro kinetic analysis of CD64-CR T cells expanded in 4 distinct healthy donors over time. After 3 days of stimulation, CD3-activated human T cells were transduced with CD64-CR. The cell surface expression of CD64 and CD3 was monitored after 7, 14, and 21 days (d) of culture at 37 °C in vitro by a PE-conjugated mouse anti-human CD64 antibody and a FITC-conjugated mouse anti-human CD3 antibody. The values are expressed as the percentage of CD3 + CD64-CR + T cells; **p < 0.01, **** *p* < 0.0001

Four distinct human CD64-CR constructs were initially evaluated. All of these proteins share extracellular CD64 and intracellular CD28/CD3ζ as signaling molecules. However, Constructs 1 and 2 included transmembrane CD28, in which the human CD64 signal peptide (SP) was replaced by a murine CD64 SP. Constructs 3 and 4 included transmembrane CD64 and CD8, respectively. The construct 1, CD64/CD28/CD3ζ (CD64-CR), was selected for our study due to its greater transduction efficiency in T cells (Fig. 1S). CD28/CD3ζ was previously shown to enhance aerobic glycolysis and reduce the expansion of classic CD19 CAR-T cells within a 2-week stimulation period in vitro [14], but its effect on CD64-CR T-cell function has not been determined. To this end, we monitored the in vitro proliferation and expansion of CD64-CR T cells for up to 4 weeks and compared them with those of CD16-CR, CD32-CR, and NT T cells in a competition assay. After 7 days of stimulation and 3 days of transduction, 95% of the cells were T lymphocytes. CD64-CR T cells exhibited significant cell expansion, outpacing the expansion of CD16-CR and CD32-CR T cells and eventually replacing NT T cells. Notably, the proportion of CD16-CR or CD32-CR T cells to total NT T cells remained constant. In contrast, CD64-CR T cells continued to expand progressively and consistently beyond the 21-day culture period to completely overcome the NT T cells (Fig. 1).

**Fig. 2:**
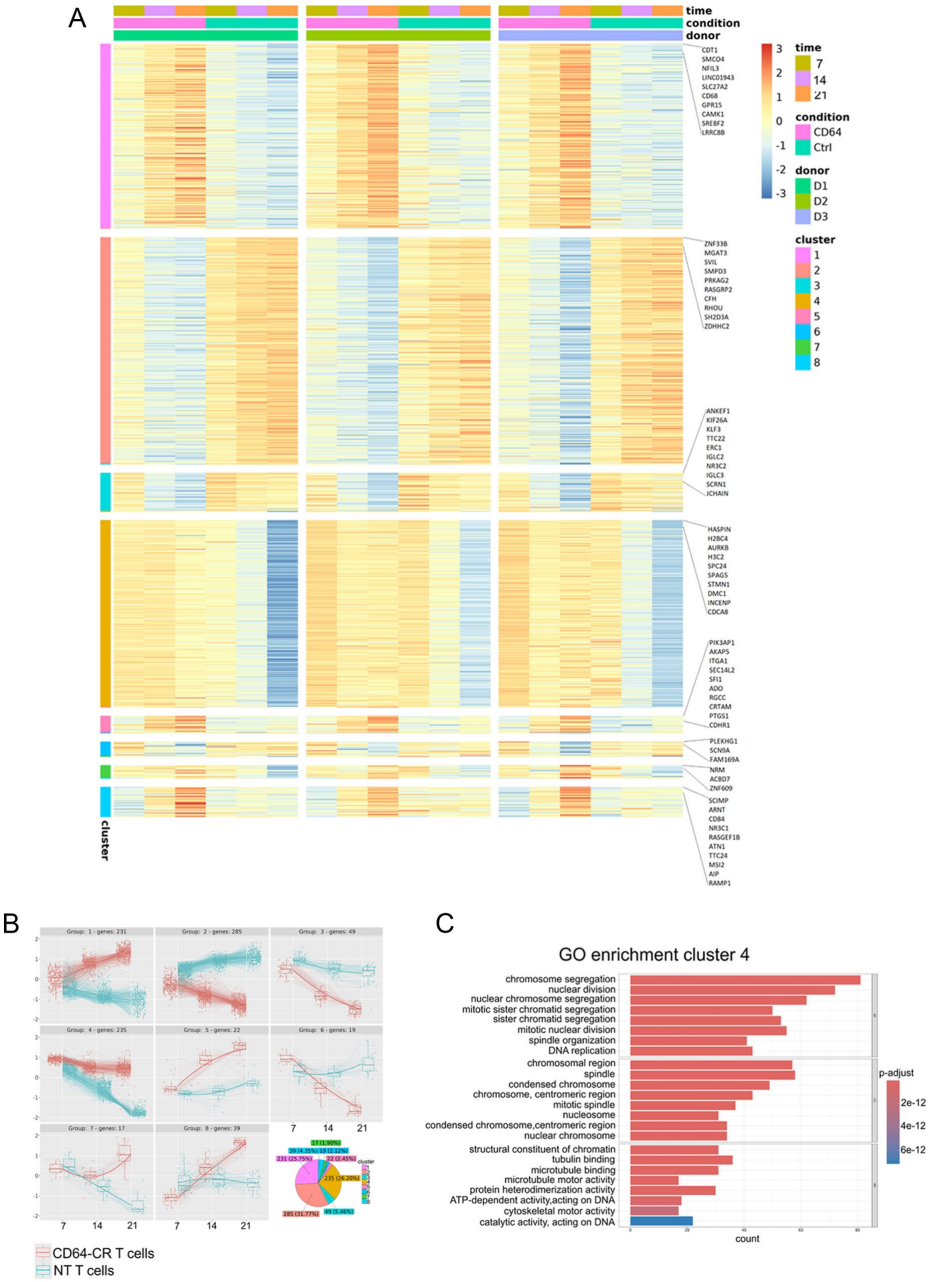
Bioinformatics analysis of transcriptomic sequencing data. (**A**) Heatmap showing gene expression levels reported as row-scaled variance stabilized data. The genes are grouped according to the previously identified expression patterns, and for each group, the name of the top 10 most statistically significant genes is shown. The annotation of the columns shows the experimental conditions, the donor, and the time point for each sample. The annotation of the lines shows the different clusters for each gene. (**B**) From the differential expression analysis conducted with DESeq2 across 3 time points and 3 donors, applying the *degPatterns* function from the ‘DEGreport’ R package, 8 distinct sets of genes that exhibited similar expression patterns were detected. The pie chart shows the distribution of genes in each of the 8 groups. (**C**) Enrichment analysis of genes belonging to cluster 4 according to the Gene Ontology (GO) biological process (BP), cellular component (CC), and molecular function (MF) categories

To investigate the genes involved in the expansion and persistence of CD64-CR T cells in vitro, we performed a comprehensive RNA sequencing (RNA-seq) analysis to compare transcriptomic differences in proliferation between CD64-CR T cells and NT T cells and correlated, enriched gene pathways. We identified 8 distinct clusters of transcriptomic profiles, including 897 differentially expressed genes. The gene expression pattern of the transduced T cells was homogeneous and time dependent, as was that of the NT cells (Fig. 2A). However, the level of gene expression in the CD64-CR T cells was distinct and inversely proportional to that in the NT T cells (Fig. 2B). Importantly, consistent with the culture data regarding cell expansion, 235 genes in cluster 4 were highly expressed and tightly linked to cell division (Fig. 2C). Interestingly, these genes were stably expressed during all incubation times in CD64-CR T cells, whereas in NT T cells, their expression rapidly decreased beginning on day 7 of incubation, after which they were progressively silenced (Fig. 2B). These data strongly suggest that CD64-CR T cells possess long-lasting proliferation and persistence ex vivo.

### The expansion of CD64-CR T cells involves reprogramming the metabolism of T cells

Immune cells shape their intracellular metabolism upon activation to meet the energetic demand necessary for cell division. In particular, dividing T cells exhibit high rates of glycolysis, which is essential for their anabolic metabolism. This metabolic remodeling is induced by TCR stimulation, which activates PI3K, Akt, Myc, mTOR, and HIF1a, among other signaling mediators [51]. Moreover, T-cell activation causes an increase in the functionality of pyruvate dehydrogenase kinase 1 (PDHK1), which halts pyruvate from entering the mitochondria and leads to the production of lactate through LDH. This step promotes the production of effector cytokines by T cells [52].

**Fig. 3:**
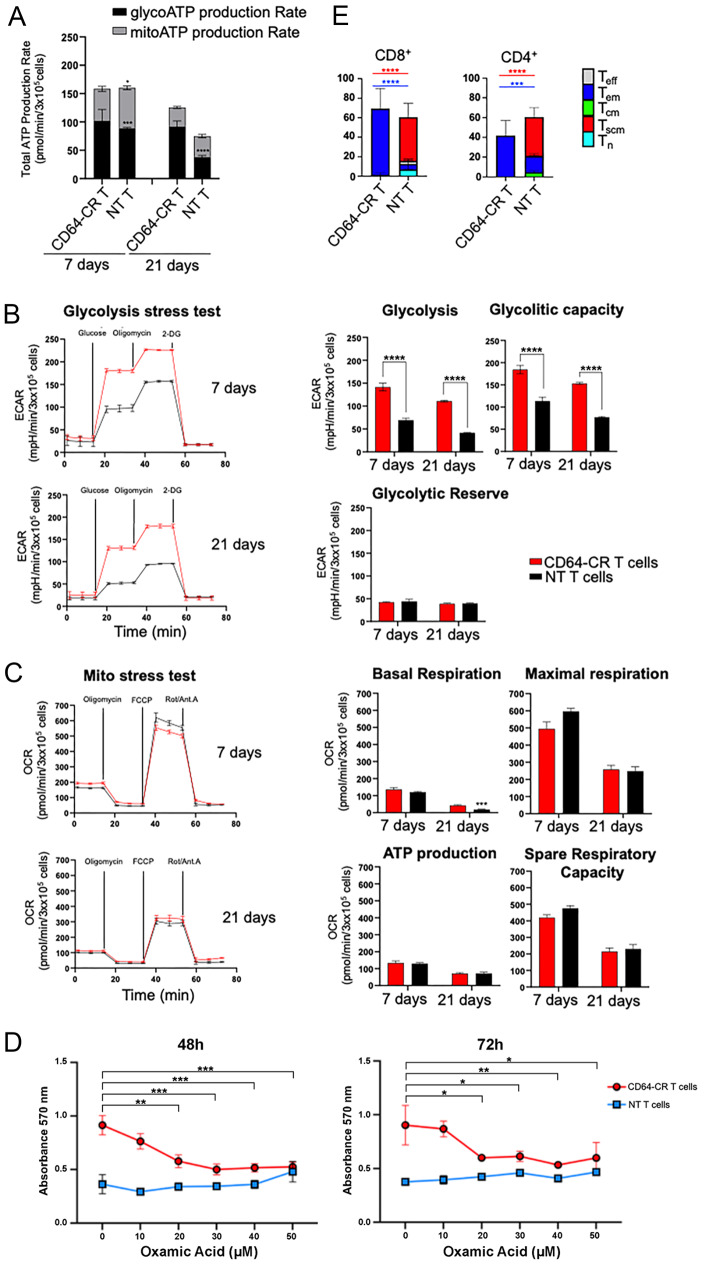
Kinetics of the mitoATP/glycoATP production rate, glycolysis and its relationship with CD64-CR T cell proliferation, and oxidative metabolism in CD64-CR T cells. (**A**) Histograms showing metabolic flux analysis indicating quantification of glycolytic ATP production and mitochondrial ATP production. (**B**) Representative extracellular acidification rate (ECAR) profile obtained through the glycolysis stress test performed on CD64-CR (red lines) and NT T (black lines) cell cultures at 7 days and 21 days after activation (left panels). The histograms show key parameters related to glycolytic function, such as glycolysis, glycolytic capacity, and the glycolytic reserve, after the addition of sequential injections of glucose, oligomycin, and 2-DG (right panels). (**C**) The left panels show a representative profile of the oxygen consumption rate (OCR) obtained through the cell Mito Stress Test. The OCR was measured after adding oligomycin, FCCP, rotenone, and antimycin A to CD64-CR (red lines) and NT T (black lines) cell cultures over time (7 days and 21 days). Histograms in the right panels show the individual parameters of basal respiration, ATP-linked respiration, maximal respiration, and spare respiratory capacity. All the data were analyzed with XFe Wave software and are expressed as the ECAR or OCR. The means ± SEMs are indicated, *p < 0.05,***p < 0.001, ****p < 0.0001, *n* ≥ 3 per group, each (n) with ≥ 12 technical replicates. *p* values were obtained using an unpaired Student’s t test. (**D**) CD64-CR T cells and NT T cells were thawed, washed twice, and resuspended in RPMI-1640 complete medium supplemented with 10 ng/ml IL-7 and 5 ng/ml IL-15 in the presence or absence of the indicated concentrations of oxamic acid. After 48 and 72 h incubation in 96 well-plates, at 37 °C, in 5% CO_2_, cells were analyzed by the MTT assay. The figure shows one representative experiment of three with similar results. The means ± SD are indicated, *p < 0.05, **p < 0.01, ***p < 0.001 (**E**) Phenotypic analysis of CD64-CR T cells after 3 weeks of activation. CD64-CR T and NT T cells from 3 healthy donors were tested for T-cell basic and differentiation markers by 9-color flow cytometry analysis. The frequencies of T naïve (Tn), T stem cell memory (Tscm), T central memory (Tcm), T effector memory (Tem), and T effector (Teff) cells in the CD8 + and CD4 + T populations are shown. The means ± SDs are indicated. Two-way ANOVA was used for statistical analysis. ***p < 0.001, ****p < 0.0001

To identify the factors affecting cell proliferation and to gather metabolic information on CD64-CR T cells and NT T cells underlying their differential expansion potential, we performed RNA-seq following TCR stimulation. Compared to those in NT T cells, 52 genes were significantly upregulated in CD64-CR cells, and these genes are associated with glycolysis/gluconeogenesis and are highlighted in red (Fig. 2S). These data suggest that, following CD3 stimulation, CD64-CR T cells preferentially reprogram their metabolism toward glycolysis. We then validated the RNA-seq results by performing functional metabolic assays. We evaluated the kinetics of the mitoATP/glycoATP production rate, glycolysis by the extracellular acidification rate (ECAR), and oxidative metabolism by the oxygen consumption rate (OCR) in CD64-CR T cells and NT T cells. The ATP rate index showed that, as expected, ATP was generated from both glycolytic and oxidative metabolism. However, the glycolytic ATP concentration was significantly greater in the CD64-CR T cells than in the NT T cells on days 7 and 21 after stimulation (Fig. 3A).

In the glycolysis stress tests, we measured the ECAR of CD64-CR T cells and NT T cells before (basal) and after the sequential addition of glucose, oligomycin, and 2-DG. CD64-CR T cells (red lines) and NT T cells (black lines) exhibited equivalent basal ECARs. However, after the addition of glucose, which stimulates basal glycolytic metabolism, the glycolytic rate of CD64-CR T cells was significantly greater than that of NT T cells on both days 7 and 21. Furthermore, the addition of oligomycin, which inhibits mitochondrial ATP production and shifts energy production to glycolysis, enhanced the glycolytic capacity of CD64-CR T cells compared to that of NT T cells, while the glycolytic reserve did not change (Fig. 3B). We then used the cell Mito Stress Test to assess the mitochondrial respiration of CD64-CR T cells and NT T cells. The basal OCR of both cell types was 2-fold lower on day 21, and the OCR was slightly lower in CD64-CR T cells than in NT T cells following serial injections of oligomycin, FCCP, or Rot/Ant. A, ATP production, maximal respiration, and spare respiratory capacity did not differ between CD64-CR T cells and NT T cells (Fig. 3C). These data indicate that CD64-CR T cells exhibit a strong increase in glycolysis consistent with their increased proliferation and, more likely, cytokine production. To demonstrate a cause-effect relationship between glycolysis and CD64-CR T cell proliferation, we examined the effects of non-toxic concentrations of oxamic acid, a lactate dehydrogenase A (LDH-A) inhibitor, on CD64-CR T cell proliferation in vitro using the MTT assay. Figure 3D shows that after 48- and 72-h incubation, oxamic acid significantly inhibited the proliferation of CD64-CR T cells in a dose-dependent manner, while NT T cells did not show similar effects. To enhance the reliability of the MTT results, we also conducted an absolute cell count of viable cells in parallel. Our findings indicate that oxamic acid significantly reduces the cell counts of CD64-CR T cells and to a lesser extend the number of NT T cells, but only at the highest concentrations. Additionally, we observed a direct correlation between the MTT optical density (OD) and the number of CD64-CR T cells. Furthermore, trypan blue exclusion analysis confirmed that at least 95% of the cells were alive under all culture conditions analyzed (Fig. 3S). These results suggest that lactate plays a role in regulating the proliferation of CD64-CR T cells.

### CD64-CR promotes T-cell differentiation toward an effector memory phenotype

**Fig. 4:**
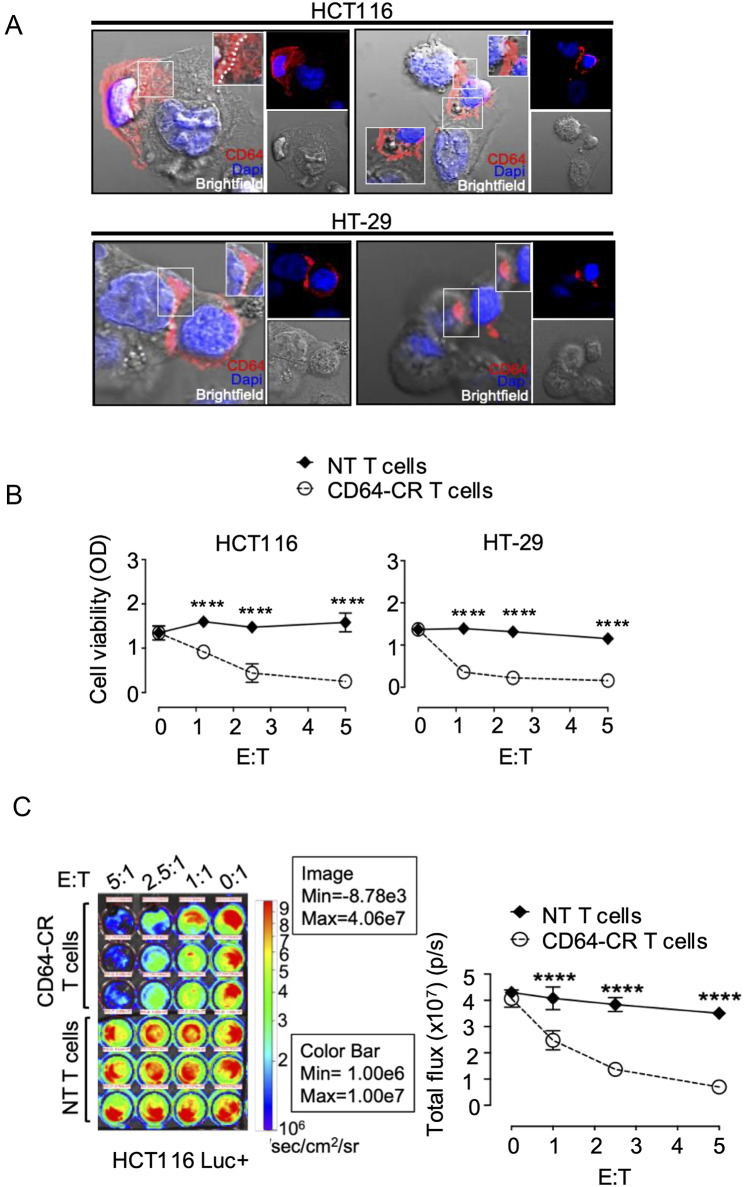
Recognition of CD64 ALs on CRC cells Triggered CD64-CR T-Cell Direct and Indirect Cytotoxicity: (**A**) CD64-CR T cells were incubated with HCT116 or HT-29 cells for 1 h at 37 °C at a 2:1 ratio. Then, the cells were indirectly immunostained with a mouse anti-human CD64 mAb (red), and the DNA was counterstained with DAPI (blue). Slides were analyzed by using a Zeiss LAM 880 confocal microscope as described in the methods section. Overlay of the staining of CD64 cell nuclei and bright field images are shown in two representative images (*n* = 3). The magnified images of single cells show the directional delivery of CD64 + T cells (red) toward the lytic synapse. The dotted line shows the cell membrane position. Confocal analysis of a single optical section is shown. (**B**) Representative MTT assay showing viable HCT116 and HT-29 tumor cells after 72 h of coculture with CD64-CR or NT T cells at different E: T ratios (*n* = 3). Each condition was tested in triplicate, and the mean ± SD of optical density (OD) measured at 570 nm is shown. Two-way ANOVA with the Bonferroni correction adjusted *p* value was used; ****p < 0.0001. (**C**) Bioluminescence (BLI) image (left panel) of HCT116 luciferase-expressing cells (HCT116 Luc+) cocultured with CD64-CR or NT T cells at different E: T ratios. Each experimental condition was tested in triplicate. Photons (total flux) emitted from HCT116 Luc + cells in the selected regions of interest (ROIs) were quantified using Living Image^®^ software (right panel). Two-way ANOVA was used for statistical analysis; ****p < 0.0001. The figure shows a representative experiment of three performed with similar results

The CD28/CD3ζ costimulatory molecule in traditional CAR constructs reprograms CAR-T-cell metabolism toward glycolysis; impairs T-cell activation, proliferation, and persistence after 14 days of in vitro culture; and promotes effector memory T-cell differentiation [14]. However, there is also evidence that Fcγ-CR and CD32/CD28/CD3ζ T cells exhibit lower levels of glycolytic genes than NT T cells. In addition, they expressed high levels of CD45RA and had a diverse cell immunophenotype, including central memory T cells (Tcm) [41]. These data suggest that CD28/CD3ζ may have heterogeneous effects on the regulation of cell metabolism and differentiation among different types of CAR-T cells. On this basis, we investigated the immunophenotypes of CD64-CR T cells and NT T cells. After 22 days of stimulation, we stained transduced and NT T cells with 9 fluorescent mAbs specific for T-cell differentiation markers. Figure 4S shows the gating strategies chosen and utilized for evaluating the flow cytometry results. Large fractions of CD8 + and CD4 + CD64-CR T cells (67.83 ± 20.63% and 41.7 ± 15.3%, respectively) were effector memory (Tem) cells (CD45RO + CCR7-CD62L-CD95 + CD27±]. Both CD8 + and CD4 + cells included a small population of CD45RO + CCR7 + or CD45RO + CD62L + cells. In contrast, the NT T cells exhibited a strictly distinct phenotype. In particular, 6.8 ± 8.9% of the CD8 + cells exhibited a T-cell naïve (Tn) phenotype (CD45RA + CCR7 + CD62L + CD95-CD27+), and 44.7%±14.23% of the cells expressed T-cell memory (Tscm) markers (CD45RA + CCR7 + CD62L + CD95 + CD27+); 5.13 ± 5.47%, Tem; and 3.13 ± 1.9, T effector [(Teff) CD45RA + CCR7-CD62L-CD95 + CD27+] (Fig. 3E, left panel). We observed similar results for the CD4 + cells (Fig. 3E, right panel). Overall, our data suggest that, despite their preferential glycolytic and Tem phenotypes, CD64-CR T cells can outperform glycolytic NT T cells in terms of expansion and proliferation, resulting in their replacement in long-term culture.

### CD64 alternative ligands on CRC cells triggered CD64-CR-dependent cytotoxicity

**Fig. 5:**
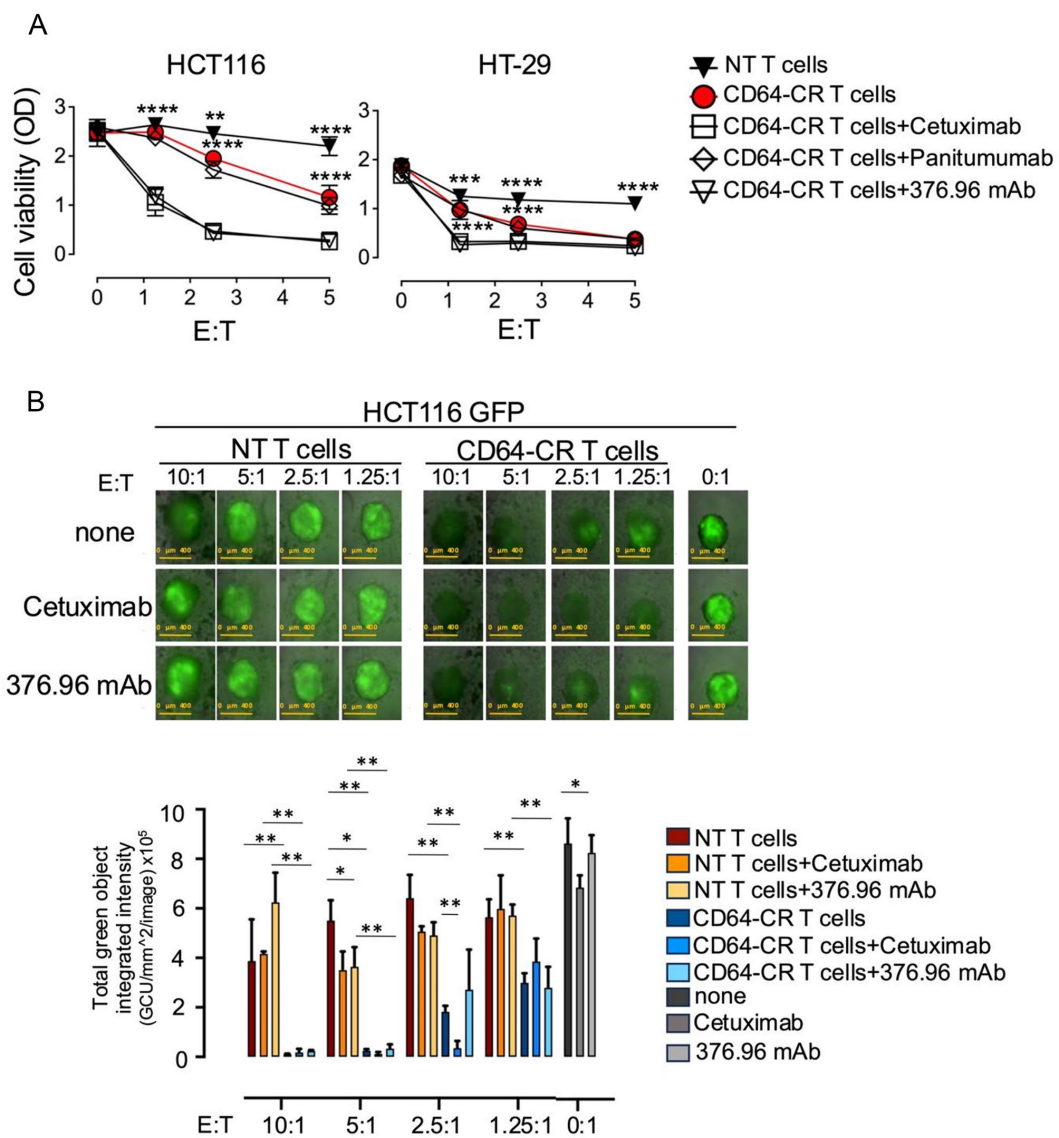
Efficient elimination of CRC cells cultured in two-dimensional (2) and three-dimensional (3D) settings by CD64-CR T-cell-dependent cytotoxicity is enhanced by mAbs specific for TAAs. (**A**) HCT116 and HT-29 cells were incubated with CD64-CR or NT T cells with or without cetuximab, panitumumab, or the anti-B7-H3 376.96 mAb at different E: T ratios. After 72 h of coculture, tumor cell viability was tested via the MTT assay. A representative graph of three independent experiments is shown; the means **±** SDs of the optical density (OD) at 570 nm are shown for triplicate samples; **p < 0.01, ***p < 0.001, ****p < 0.0001. (**B**) HCT116 cells transduced with lentivirus to express green fluorescent protein (GFP) were cultured for 72 h in ultralow adherence cell plates to allow 3D spheroid formation. Then, CD64-CR T or NT T cells were added to HCT116 GFP 3D spheroids at different E: T ratios in the presence or absence of the indicated antibodies. The effect of T cells on HCT116 3D spheres was visualized by fluorescence imaging (upper panels). Images were captured using an IncuCyte Live-Cell Analysis System. The total integrated fluorescence intensity within the cell spheroids per image was calculated using IncuCyte Software (lower panel). The mean ± SD of triplicate samples of one of two experiments is shown. An unpaired t test was used for statistical analysis; *p < 0.05, **p < 0.01

**Fig. 6:**
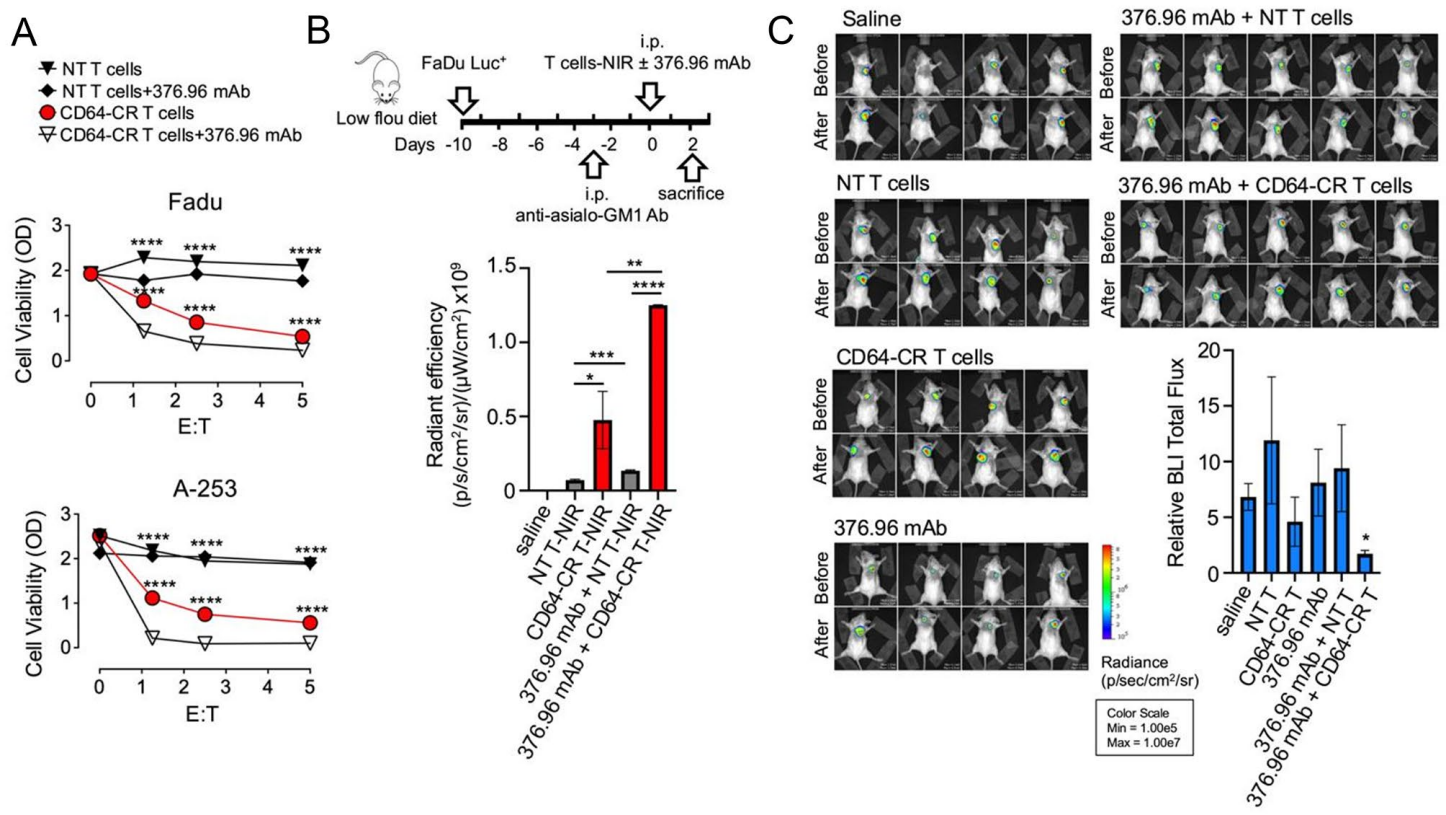
CD64-CR T cells mediated direct and indirect cytotoxicity in SCCHN cell lines in vitro, while only indirect cytotoxicity was effective in vivo. (**A**) An MTT cell viability assay was performed on SCCHN FaDu and A-253 cells after 72 h of culture with CD64-CR or NT T cells in the presence or absence of 376.96 mAb at the indicated E: T ratios. The data shown are representative of three independent experiments; mean ± SD of triplicate samples. Two-way ANOVA was used for statistical analysis; ****p < 0.0001. (**B**) Upper panel: Schematic representation of the tracking of CD64-CR T cells in subcutaneous SCCHN xenografts generated from CB-17 SCID mice. Lower panel: FaDu Luc^+^ cells (5.0 × 10^5^) were implanted in the dorsal subcutaneous area of CB17-SCID mice. After 10 days, the animals were divided into 5 groups of three and treated with T cells NIR with or without 150 μg of the mAb 376.96, as indicated in the lower panel. After 48 h, the mice were sacrificed. Then, the tumor was removed, and fluorescence imaging was performed (see MM). The data represent the cumulative numbers of three tumors in each of the five groups of CB17-SCID mice analyzed: *p < 0.05, **p < 0.01, ***p < 0.001, and ****p < 0.0001. (**C**) To assess the antitumor activity of CD64-CR T-cell treatment in vivo, FaDu Luc^+^ cells (5.0 × 10^5^) were subcutaneously inoculated into the dorsal region of CB17-SCID mice. Animals were randomly divided into 6 groups (*n* = 4–5/group as indicated): 12 (1st dose) and 24 (2nd dose) days after tumor cell injection. The following treatments were administered by intraperitoneal injection: group 1, saline; group 2, 2.5 × 10^6^ NT T cells; group 3, 2.5 × 10^6^ CD64-CR-transduced T cells; group 4, 150 μg of 376.96 mAb; group 5, 150 μg of 376.96 mAb; and after 1 h, 2.5 × 10^6^ NT T cells; group 6, 150 μg of 376.96 mAb; and after 1 h, 2.5 × 10^6^ CD64-CR T cells. Tumor growth was assessed using bioluminescence imaging (BLI) before and after the treatments. The column bars indicate the tumor BLI emission in each group measured after the second T-cell administration relative to the corresponding values assessed before the treatments. In both B and C, the values are expressed as the mean ± SEM; * *p* < 0.05 vs. control values. The figure presents a comparison of each animal’s condition before and after treatment, with the relative luminescence displayed in the graph

Recently, we showed that extracellular CD32 (CD28/CD3ζ)-reactive T cells recognize ALs and trigger HLA-unrestricted cytotoxicity in a subset of BC cells, including triple-negative breast cancer (TNBC) cells [41]. Thus, we hypothesized that cancer cells may also express CD64 ALs. CD64-CR T cells were indirectly stained with anti-CD64 mAbs (red) and incubated with HCT116 or HT-29 CRC cells. CD64-CR polarized at the cell-to-cell contact interface, leading to immunological synapse formation and directional delivery of CD64 granules (red) toward both HCT116 and HT-29 cells (Fig. 4A). An animation of CD64-CR polarization at the immunological synapsis level is also shown in Fig. 5S. To assess the anticancer activity of these combinations, 14-day cultured CD64-CR T cells and NT T cells were incubated with either HCT 116 or HT-29 cells at different E: T ratios for 72 h at 37 °C. Figure 4B shows that CD64-CR T cells significantly affected the viability of both HCT116 and HT-29 cells, even at very low E: T ratios, whereas NT T cells did not. To demonstrate that the anticancer effect of engineered T cells in vitro was due to direct cytotoxic activity rather than antiproliferative activity, we performed a flow cytometry-based cytotoxicity assay utilizing annexin V/PI staining. CD64-CR T cells at an E: T ratio of 2:1 induced either apoptosis or transition to necrosis in CRC cells (Fig. 6S A and B). We then assessed the persistence of the anticancer effect of CD64-CR T cells beyond day 14. After 21 days of incubation, in vitro bioluminescence imaging (BLI) analysis was performed on the CD64-CR T cells. CD64-CR or NT T cells and firefly luciferase-expressing HCT116 Luc + cells were incubated at different E: T ratios for 72 h, as indicated, at 37 °C. The signal produced by HCT116 Luc + was visualized by BLI. Consistent with the data from 14-day cultures, a significant reduction in the number of luminescent cells was detected in the presence of CD64-CR T cells, while NT T cells were ineffective (Fig. 4C left and right panels). Our results indicate that the surface expression of CD64 ALs in cancer cells increases susceptibility to CD64-CR T-cell-dependent cytotoxicity in vitro [40, 41]. Furthermore, they also provide evidence of the sustained, long-lasting anticancer cytotoxic potential of CD64-CR T cells *in vitro.*

### Direct CD64-CR T cell-mediated cytotoxicity is enhanced by ADCC in 2D and 3D culture systems in vitro

Next, we explored the connection between CD64-CR-T-cell-dependent cytotoxicity and the ADCC. Engineered T and NT T cells, either alone or with anti-EGFR mAbs such as cetuximab (IgG1), panitumumab (IgG2) or the anti-B7-H3 mAb 376.96 (IgG2), were cultured with or without EGFR + B7-H3 + cells for 72 h in vitro. CD64-CR T cells killed HCT116 cells. Interestingly, cetuximab or the 376.96 mAb enhanced CD64-CR-T-cell cytotoxicity against HCT116 cells, while panitumumab did not, as expected. We obtained similar results when HT-29 CRC cells were utilized; although they appeared less sensitive to the combination of CD64-CR T cells with mAbs than did HCT116 cells. In contrast, neither the NT T cells nor the anti-EGFR mAbs or the anti-B7-H3 mAb affected the viability of the HCT116 or HT-29 CRC cells (Fig. 5A). In vitro immune cell interactions with cancer cells are typically studied using two-dimensional (2D) culture systems. However, 2D systems have limitations, including restricted cell-to-cell contact and a lack of gradient diffusion [53, 54]. Conversely, three-dimensional (3D) culture systems mirror the TME more faithfully than 2D systems since they retain dominant cell–cell contacts [55], mimic cancer cell growth [56], and allow gradient diffusion [57].

Based on this information, we carried out a study to assess the effects of CD64-CR T cells on 3D CRC spheroids. NT T cells or CD64-CR T cells were incubated with spheroids green fluorescent protein (GFP)-containing HCT116 in the presence or absence of cetuximab or the 376.96 mAb at different E: T ratios. After 72 h of incubation at 37 °C, in vitro, CD64-CR T cells induced complete elimination of green fluorescence at the highest E: T ratios and partial clearance at lower E: T ratios. However, NT T cells with or without targeting mAs did not affect spheroid fluorescence, independent of the mAbs utilized. Interestingly, the combination of cetuximab with engineered T cells led to the complete elimination of spheroid green fluorescence at any E: T ratio, while the combination of CD64-CR T cells with the 376.96 mAb was less efficient but still induced a substantial impact on spheroid fluorescence (Fig. 5B). These data suggest that CD64-CR T cells are powerful cytotoxic cells capable of mediating both HLA-unrestricted cytotoxicity and ADCC and can easily eliminate CRC cells in 2D and 3D culture systems.

### CD64-CR T cells identify alternative ligands of CD64 on SCCHN cells while triggering antibody-dependent cellular cytotoxicity in vitro and in vivo

The next question was whether the recognition of CD64 ALs was restricted to CRC cells or could be extended to additional cancer types. Moreover, CD64-CR T cells can recognize ALs on SCCHN cells, such as FaDu and A253. Figure 6A shows that CD64-CR T cells specifically affected the viability of both FaDu and A253 cells at an equal E: T ratio. Moreover, adding the 376.96 mAb to CD64-CR T cells reduced the viability of both cancer cell lines. However, the presence or absence of the 376.96 mAb in combination with NT T cells did not impact the viability of the target cells. These results suggest that CD64-CR recognizes CD64-CR ligands on SCCHN cells, leading to NK-like cytotoxicity involving 2 distinct cytotoxic functions in vitro, direct cytotoxicity and ADCC.

The antitumor activity of CD64-CR T cells was also investigated in vivo utilizing a xenograft mouse model (Fig. 6B, upper panel). We first assessed the biodistribution of CD64-CR T cells or NT T cells in the presence or absence of the 376.96 mAb in subcutaneous tumors obtained from CB17-SCID mice as indicated in Fig. 6B, lower panel. Then, we assessed their antitumor activity. Thus, six groups of CB17-SCID mice were injected subcutaneously into the dorsal region with 5 × 10^5^ FaDu Luc + cells. Twelve and 24 days after tumor cell injection, the mice were intraperitoneally inoculated with NT or CD64-CR T cells with or without the 376.96 mAb as indicated (Fig. 6C). Tumor growth was monitored by BLI before and after the treatments, and the photons emitted by the tumor mass were quantified (Fig. 6C). Our data indicate that, despite the tumors obtained from mice injected with CD64-CR T cells, with or without the anti-B7-H3 mAb, emitted significantly higher fluorescence than that of tumor controls, only the combination of CD64-CR T cells with the 376.96 mAb significantly inhibited tumor mass growth. These results suggest a preferential infiltration of tumor mass by CD64-CR T cells but the antitumor activity observed involved an ADCC-dependent mechanism. Furthermore, additional studies will be necessary to understand and optimize the antibody-independent antitumor activity of CD64-CR T cells in vivo.

### CD64-CR upregulates PD-L1 and HLA-DR in CRC and SCCHN carcinoma cells

Upregulation of PD-L1 enhances the susceptibility of cancer cells to the combination of CD64-CR and atezolizumab through specific production of IFNγ.

**Fig. 7:**
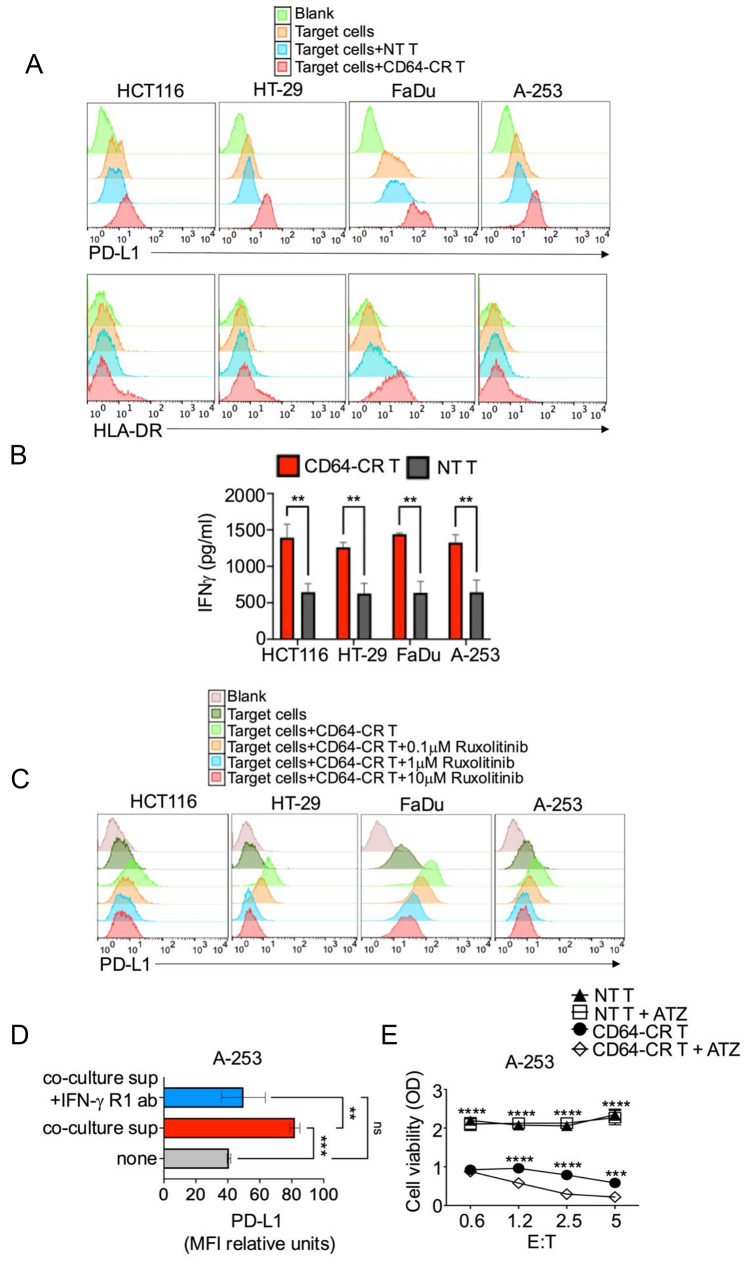
CD64-CR T cells modulated PD-L1 and HLA-DR expression in CRC and SCCHN cells via IFNγ. (**A**) Histogram plots showing PD-L1 (upper panels) and HLA-DR (lower panels) surface expression in CRC and SCCHN patients. As indicated, the cancer cells were incubated in the presence or absence of NT T cells or CD64-CR T cells for 24 h at an E: T ratio of 1:1. Then, the NT T cells and CD64-CR T cells were removed, and the cancer cells were stained with PE-conjugated anti-human PD-L1 or anti-HLA-DR mAbs and analyzed by flow cytometry. The data are representative of 3 independent experiments. (**B**) CD64-CR T cells specifically induced high levels of IFNγ production. Just before target cell staining for PD-L1 was performed, supernatants were collected from the cell culture conditions described in panel A. IFNγ was quantified via an ELISA. The mean values of two independent experiments are shown. The error bars indicate the SDs; **p < 0.01. (**C**) The Janus kinase inhibitor (JAK) ruxolitinib inhibited CD64-CR-T-cell-dependent PD-L1 upregulation in CRC and SCCHN cells. Cancer cells were incubated in vitro at 37 °C for 3 h with scalar doses of ruxolitinib with or without CD64-CR T cells (E: T 1:1) as indicated. Then, the cancer cells were stained with an anti-PD-L1 Ab, as described in panel A, and analyzed by flow cytometry. A representative of three independent experiments is shown. (**D**) PD-L1 overexpression is regulated by IFNγ-induced CD64-CR T cells. A-253 cells were cultured with or without spent supernatants (sup) from A-253 cells incubated at 37 °C in vitro for 24 h in the presence or absence of CD64-CR T cells and with or without saturating doses of the anti-IFNγR1 mAb. Then, the expression of PD-L1 on A-253 cells was assessed by flow cytometry. The relative mean fluorescence intensity (MFI) is shown. The mean ± SD of three independent experiments is indicated. ns = not statistically significant, **p < 0.01, ***p < 0.001. (**E**) Atezolizumab (ATZ) increased CD64-CR-related T-cell cytotoxicity against A-253 cells. NT T cells or CD64-CR T cells were incubated with A-253 cells in the presence or absence of 100 ng/ml ATZ. After 72 h, A-253 cell viability was assessed via MTT. The results are representative of three independent experiments and are shown. The mean ± SD of three technical replicates is shown. Two-way ANOVA with Bonferroni correction was used for statistical analysis; ***p < 0.001, ****p < 0.0001

The type and functions of immune cells that infiltrate the TME play critical roles in the development of cancer [58]. Their activation or exhaustion may be steered by markers expressed on cancer cells, which often correlate with clinical outcomes. Despite PD-L1 being considered a negative prognostic factor in solid cancers, in SCCHN and CRC [58], it is a favorable prognostic marker similar to HLA-DR [59]. Thus, we tested the relationship between CD64-CR T cells and the expression of PD-L1 and HLA-DR in both CRC and SCCHN cells. Figure 7A shows that following a 24-hour incubation with the indicated cell lines, the cancer cells were PD-L1 + and HLA-DR- except for the FaDu cells. Interestingly, in the presence of CD64-CR T cells, PD-L1 was upregulated in both CRC and SCCHN cells, while HLA-DR was upregulated only in FaDu cells. Additionally, the latter was slightly expressed *de novo* in a fraction of HCT116 and HT-29 cells (Fig. 7A, lower panels). In addition, in the presence of cancer cells, CD64-CR T cells specifically produced high levels of IFNγ (Fig. 7B). To investigate the role of IFNγ in PD-L1 upregulation, we first incubated cancer cells without or with CD64-CR T cells in the presence or absence of a scalar dose of the JAK2 inhibitor ruxolitinib, which inhibited the upregulation of PD-L1 in a dose-response manner (Fig. 7C). To provide evidence that IFNγ is directly responsible for the upregulation of PD-L1, A-253 SCCHN cells were incubated at 37 °C without or with a 24-h culture supernatant obtained from the incubation of CD64-CR T cells with A-253 cells.

Figure 7D clearly shows that the culture supernatants significantly upregulated PD-L1, which was significantly inhibited in the presence of a mAb specific for the IFNγR1 receptor. Interestingly, incubation of CD64-CR T cells in combination with atezolizumab enhanced the direct cytotoxicity of CD64-CR T cells toward A-253 cells (Fig. 7E). These results provide evidence of the immunoregulatory function of IFNγ-induced CD64-CR T cells.

## Discussion

Due to their potential clinical relevance, CD64-CR T cells represent the subject of active research efforts. In recent studies, CD64/CD3ζ-engineered NK-92 cells were successfully redirected by a combination of mAbs to target cancer cells [60]. In addition, 3 distinct extracellular Fcγ-CRs, CD16, CD32, and CD64, were fused to CD28/4-1BB/CD3ζ and tested for their anticancer activity. In combination with mAbs, CD64-CR exerts significant antitumor effects [61]. More recently, in combination with mAbs, CD64/4-1BB CR T cells were shown to have greater tumor killing efficacy and longer persistence (up to day 9 of culture) than did standard CD16-CR T cells and CD32-CR T cells [62]. These studies have shown the ability of Fcγ-CR T cells to trigger ADCC in a variety of cancer cells both in vitro and in vivo, where costimulatory signals play a crucial role. However, none of these studies have considered the impact of CD28/CD3ζ signaling on CD64-CR T-cell functions, and the ability of these signaling pathways to mediate antibody-independent cytotoxicity has not been explored [63].

We first evaluated the cell expansion capacity and metabolic phenotype of CD64-CR T cells. Then, we investigated the existence of CD64 ALs, the direct (in the absence of ADCC), and indirect (in the presence of ADCC) targeting of CRC and SCCHN cells. Finally, we explored the immunoregulatory functions of CD64-CR during CD64-CR-T-cell conjugation with cancer cells. Compared to the 4-1BBζ costimulatory molecule, the costimulatory molecule CD28/CD3ζ promoted the metabolic switch of classic CAR-T cells toward a glycolytic phenotype and effector T-cell differentiation, resulting in reduced cell expansion and persistence of CAR-T cells in vitro [14]. However, its role in CD64-CR-related T-cell proliferation, expansion, metabolic switching, and differentiation has not been explored. In agreement with the literature, CD64-CR T cells exhibited predominant glycolytic phenotypes and effector memory differentiation. However, despite these findings, CD64-CR T cells maintained a high proliferative capacity, ease of expansion, and long persistence in vitro, even after 28 days of culture. Despite CD16-CR, CD32-CR, and CD64-CR T cells sharing the CD28/CD3ζ signaling domain, CD64-CR cells exhibited the highest cell expansion profile. After up to four weeks of culture, the CD64-CR T cells were close to replacing the NT T cells. Notably, the proliferative capacity of CD64-CR T cells was associated with persistent overexpression of hundreds of genes involved in the regulation of cell proliferation beyond 21 days of culture, while the expression of those genes was rapidly downregulated in NT T cells.

These findings suggest that, at least for Fc-γCR T cells, CD28/CD3ζ may differentially regulate the proliferation, expansion, and persistence of engineered T cells. To support our thesis, we recently showed that CD32-CR T cells are characterized by lower expression of hallmark genes associated with glycolysis than are NT T cells. Conversely, CD64-CR T cells demonstrated significantly greater glycolytic activity than did NT T cells. Additionally, after 3 weeks of stimulation, CD64-CR T cells exhibited preferential differentiation toward Tem cells, whereas CD32-CR T cells displayed heterogeneous cell differentiation, with a predominant representation of Tcm cells. The metabolic and proliferative gene expression, cell expansion, and differentiation of CD64-R T cells are distinct from those of CD32-CR T cells [41]. These results strongly suggest that the regulation of Fcγ-CR T-cell functions by CD28/CD3ζ might differentially impact the proliferation, expansion, persistence, metabolism, and differentiation of Fcγ-CR T cells in vitro, depending on the type and quality of the extracellular FcγR expressed by the chimeric receptor. Alternatively, differences in transmembrane molecules, such as CD8a for CD16 and CD32-CR and CD28 for CD64-CR, may play a functional role. Further studies, including the generation of Fcγ−CR constructs sharing identical transmembrane domains, are warranted to explore this issue.

To demonstrate the presence of cell surface CD64 ALs in cancer cells, we used CD64-CR T cells either as biosensors or as cytotoxic effector cells, as we did to detect CD32 ALs in BC cells in vitro [41]. In this setting, the T-cell killing machinery is activated when the extracellular Fcγ-CR domain binds to its ligand, thereby promoting the mobilization of intracellular cytotoxic granules toward immunological synapses, leading to killing of the target cells [41]. Importantly, wild-type CD16A was also previously shown to mediate cytotoxicity upon direct interaction with undefined ALs [40]. In addition, some murine studies have shown that pre-T cells express FcγR on the cell surface during early fetal thymic development [64]. This evidence underlines the potential role of FcγRs as molecules implicated in direct cell‒cell interactions. Similarly, CD16 and CD32 were observed during murine B-lineage progenitor development [65]. While CD16A and CD32 recognition of ALs on malignant cells has been successfully demonstrated in humans, no information is presently available for CD64. Thus, we can speculate that if cancer cells express CD64 ALs distinct from those expressing CD32 or CD16, this finding represents evidence supporting the existence of an interesting group of targetable tumor antigens. In this context, Fcγ-CR T cells could target cancer cells by two distinct cytotoxic mechanisms, HLA-unrestricted cytotoxicity and ADCC, in combination with mAbs specific for known TAAs. Indeed, we present data supporting the expression of CD64 ALs on the surface of a variety of CRC and SCCHN cell lines. Upon conjugation of CD64-CR T cells with CRC cells, CD64-CR cells polarized at immunological synapses, resulting in CRC and subsequent elimination of SCCHN in vitro in an HLA-unrestricted manner. The cytotoxic effect of CD64-CR T cells was potent and efficient even at an effector/target ratio less than 1:1. Interestingly, the direct cytotoxicity of CD64-CR T cells was significantly increased by treatment with cetuximab or the anti-B7-H3 antibody and by treatment with 376.96 mAb against EGFR-positive HCT116 cells and, to a lesser extent, against HT-29 CRC cells. In contrast, panitumumab (IgG2a) had no effect on these parameters. Furthermore, the anticancer activity of CD64-CR T cells was also demonstrated in a 3D setting, in which the T cells better resembled the TME than did those in the 2D setting. However, although the literature suggests that IgG2 antibodies do not bind CD64 [66], the direct cytotoxicity of CD64-CR T cells appears to be enhanced by the anti-B7-H3, 376.96 (IgG2) mAb both in vitro and in vivo. CD64-CR T cells, in combination with the mAb 376.96, inhibited the growth of subcutaneous SCCHN FaDu cells in immunodeficient mice. We do not have a clear explanation, but for sure, the 376.96 mAbs and panitumumab have different origins—mice and humans. On the other hand, we failed to detect the anticancer activity of CD64-CR T cells in vivo in the absence of the 376.96 mAb. While experimental procedures might need to be optimized, the hostile TME is known to display a variety of evasion mechanisms favoring tumor growth, anatomical barriers preventing immune cell infiltration, glycolytic metabolism inhibiting cell expansion and persistence, and immunosuppressive cells promoting an anti-inflammatory milieu. Notably, cell distribution studies indicated that although both transduced and NT T cells traveled through the tumor, CD64-CR T cells, alone or in combination with the mAb 376.96, showed a significantly greater rate of cancer-related homing than did the controls. These data suggest that the CD64-CR-related T-cell infiltration potential might be further optimized in future studies. Moreover, a negative impact of cancer cell aerobic glycolysis on CD64-CR T-cell functions is unlikely since these cells exhibit optimal cell expansion, proliferation, persistence, and dual anticancer activity in a glycolytic microenvironment in vitro.

Nevertheless, CD64-CR T cells can act as universal CAR-T cells as long as an anti-TAA mAb is available. Our data showed that in addition to augmenting ADCC, CD64-CR-dependent cytotoxicity boosted the synthesis of IFNγ, which in turn specifically induced the upregulation or *de novo* expression of key immunoregulatory cell surface molecules, including PD-L1, in CRC and SCCHN cells and HLA-DR preferentially in FaDu cells but also in subsets of SCCHN and CRC cells. The binding of PD-L1 to PD-1 in the TME induces exhaustion of T cells, enhancing the chances of cancer evasion. However, the expression of PD-L1 is also associated with a more favorable clinical course in CRC and SCCHN [67], whereas in a recent meta-analysis, progression-free survival improved in SCCHN patients with PD-L1 expression [67]. We found that in the absence of the TAA-specific mAb, CD64-CR T cells produce high amounts of IFNγ, which in turn promotes the expression of PD-L1 and HLA-DR on tumor cells. Most importantly, the addition of a therapeutic anti-PD-L1 mAb to cocultures resulted in high ADCC activity. These results are potentially clinically relevant since they indicate that mAb-independent and mAb-dependent antitumor mechanisms elicited by CD64-CR T cells could have additional anticancer effects. Moreover, they suggested that CD64-CR T cells could complement standard treatments with mAbs inhibiting immunological checkpoints by targeting tumor cells expressing these markers. Similarly, HLA-DR expression by tumor cells could help build up and improve the antitumor activity of resident T cells. Indeed, the expression of HLA class II has previously been shown to be associated with improved survival and a more favorable clinical course of CRC [59].

The findings presented in this study, along with information from existing literature, suggest that CD64-CR T cells similarly to CD32-CR T cells have significant potential for use in the design of personalized clinical studies. In addition, a recent Phase I trial demonstrated that CD16-CR T cells, when administered alongside rituximab to patients with relapsed or refractory CD20-positive B cell lymphoma, were tolerated well with limited toxicity [24]. Nonetheless, two important caveats need to be considered before pursuing a similar therapeutic approach. First, the molecular nature of the ALs associated with the FcγCR, including CD64-CR, remains unknown. Second, future studies must address the expression and distribution of ALs in normal cells. Nevertheless, even if the identification of ALs in normal human cells poses limitations on the clinical design of CD64-CR T cell-based immunotherapy, it could offer valuable insights into the role and function of FcγR in human cell biology.

## Electronic supplementary material

Below is the link to the electronic supplementary material.

Supplementary Material 1

Supplementary Material 2

## Data Availability

All the data in this study are available in the manuscript and supplementary information. The Illumina RNA-Seq raw reads from T cells were submitted to the NCBI SRA database under BioProject PRJNA1171197.

## Abbreviations

2-DG: 2-Deoxy-D-glucose
ADCC: Antibody-dependent cellular cytotoxicity
ALs: Alternative ligands
BLI: Bioluminescence/Bioluminescent Imaging
CAR: Chimeric antigen receptor
CR: Chimeric receptor
CRC: Colorectal cancer
ECAR: Extracellular acidification rate
EGFR: Epidermal growth factor receptor
ELISA: Enzyme-linked immunosorbent assay
FCCP: Carbonyl cyanide 4-(trifluoromethoxy)phenylhydrazone
FcγR: Fc gamma receptor
FLI: Fluorescence imaging
GFP: Green fluorescent protein
HLA: Human leukocyte antigen
IFNγ: Interferon gamma
IL-15: Interleukin-15
IL-7: Interleukin-7
Luc: Luciferase
mAb: Monoclonal antibody
NIR: Near-infrared
NT: Non-transduced
OCR: Oxygen consumption rate
OD: Optical density
PD-L1: Programmed death-ligand 1
PI: Propidium iodide
SCCHN: Squamous cell carcinoma of the head and neck
SCID: Severe combined immunodeficiency disease
TAA: Tumor-associated antigen
TME: Tumor microenvironment
