# Supplementary material for: The CD64/CD28/CD3ζ chimeric receptor reprograms T-cell metabolism and promotes T-cell persistence and immune functions while triggering antibody-independent and antibody-dependent cytotoxicity
Source: Exp Hematol Oncol. 2025 Feb 17;14:17. doi: 10.1186/s40164-025-00601-2 (PMC11834217; doi:10.1186/s40164-025-00601-2)
Supplement: Supplementary file 1 — Supplementary Material 1 [file 40164_2025_601_MOESM1_ESM.docx]

**Supplementary Figures**

**
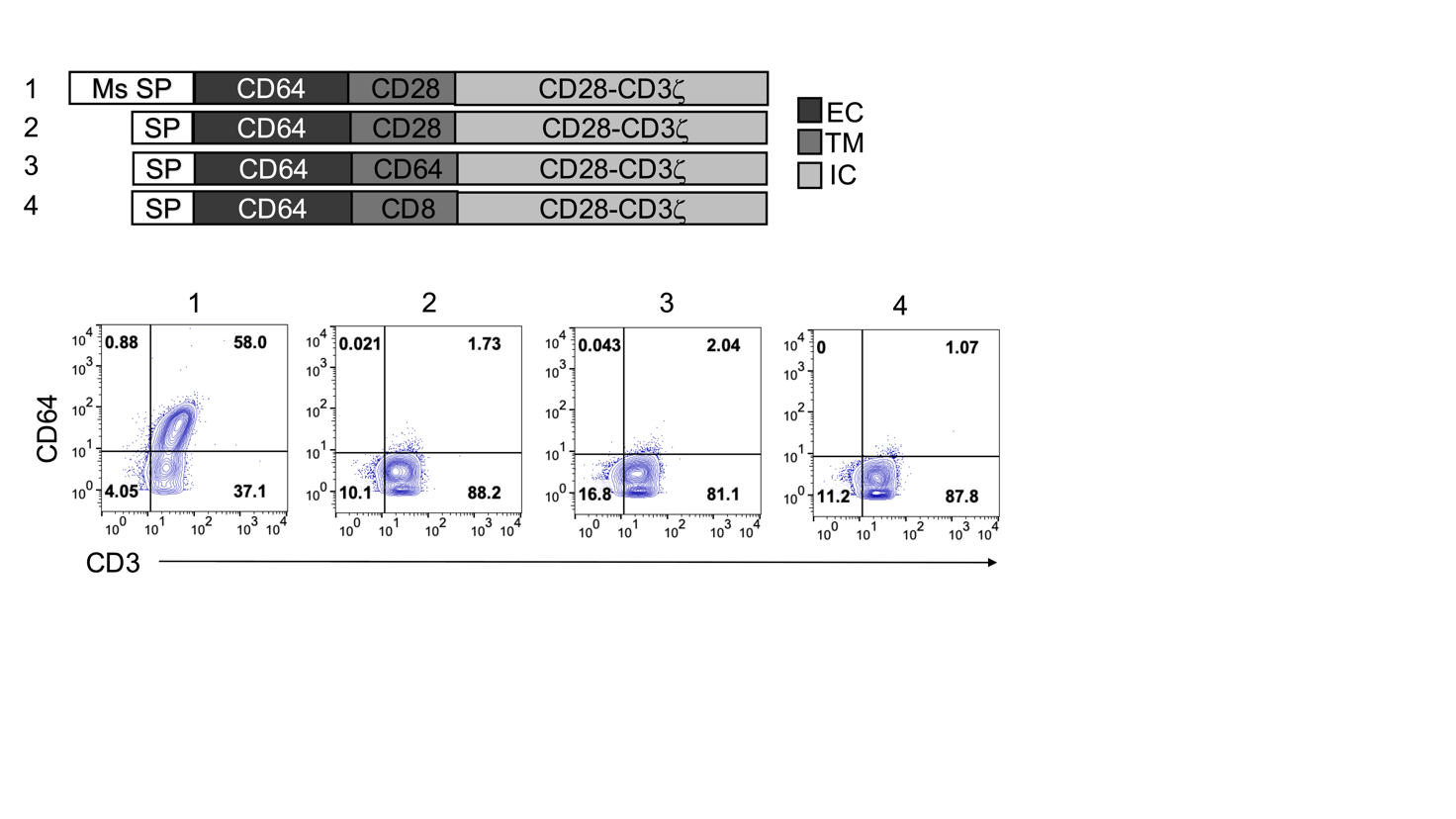
**

**Figure 1 S.**

The upper panel shows the schematic representation of CD64-CR constructs. All CD64 chimeric constructs (1-4) shared the extracellular region of the human CD64 and the intracellular signaling motifs CD28/CD3ζ. Constructs 1 and 2 included CD28 transmembrane domain while constructs 3 and 4 had CD64 and CD8 transmembrane domains, respectively. Construct 1 carried a CD64 mouse leader sequence. Ms SP= CD64 mouse signal peptide, SP= CD64 human signal peptide, EC= extracellular, TM: transmembrane, IC: intracellular. The lower panel shows the analysis of the surface expression of the four constructs. T lymphocytes were engineered with the indicated constructs, stained with the anti-human CD3 and anti-human CD64 antibodies, and analyzed by flow cytometry. Ten thousand events were acquired for each sample. Contour plots are representative of three independent experiments.

**
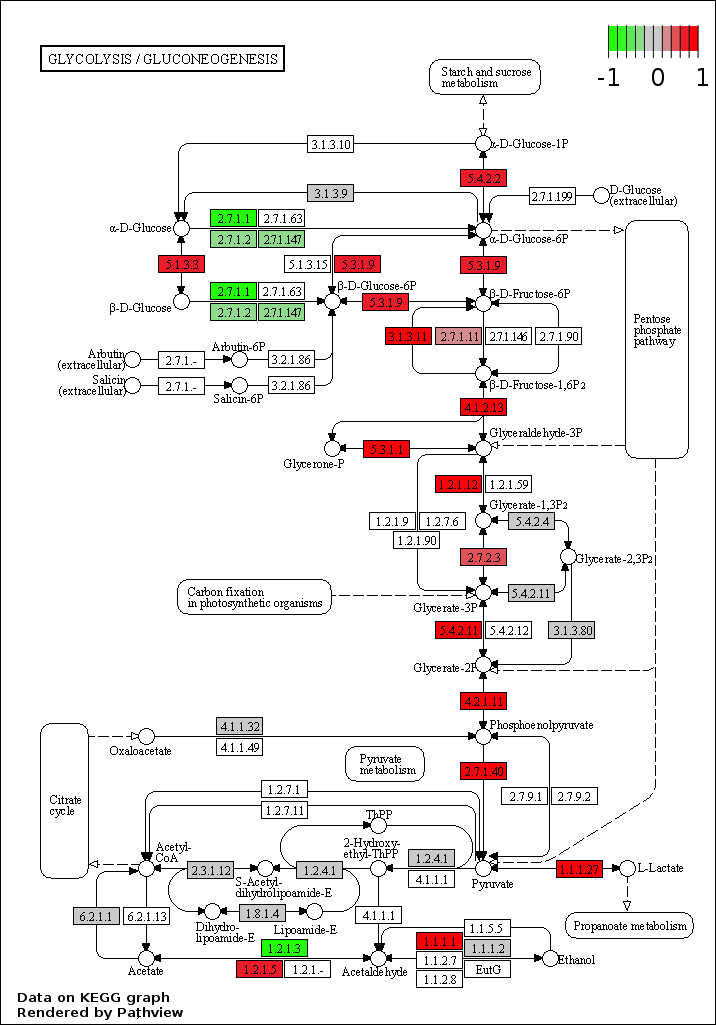
**

**Figure 2 S.**

Pathview KEGG plot showing the expression levels as fold changes of the genes coding for the main enzymes or enzymatic complexes involved in the Glycolysis/Gluconeogenesis metabolic pathway of CD64-CR T cells versus NT T cells.


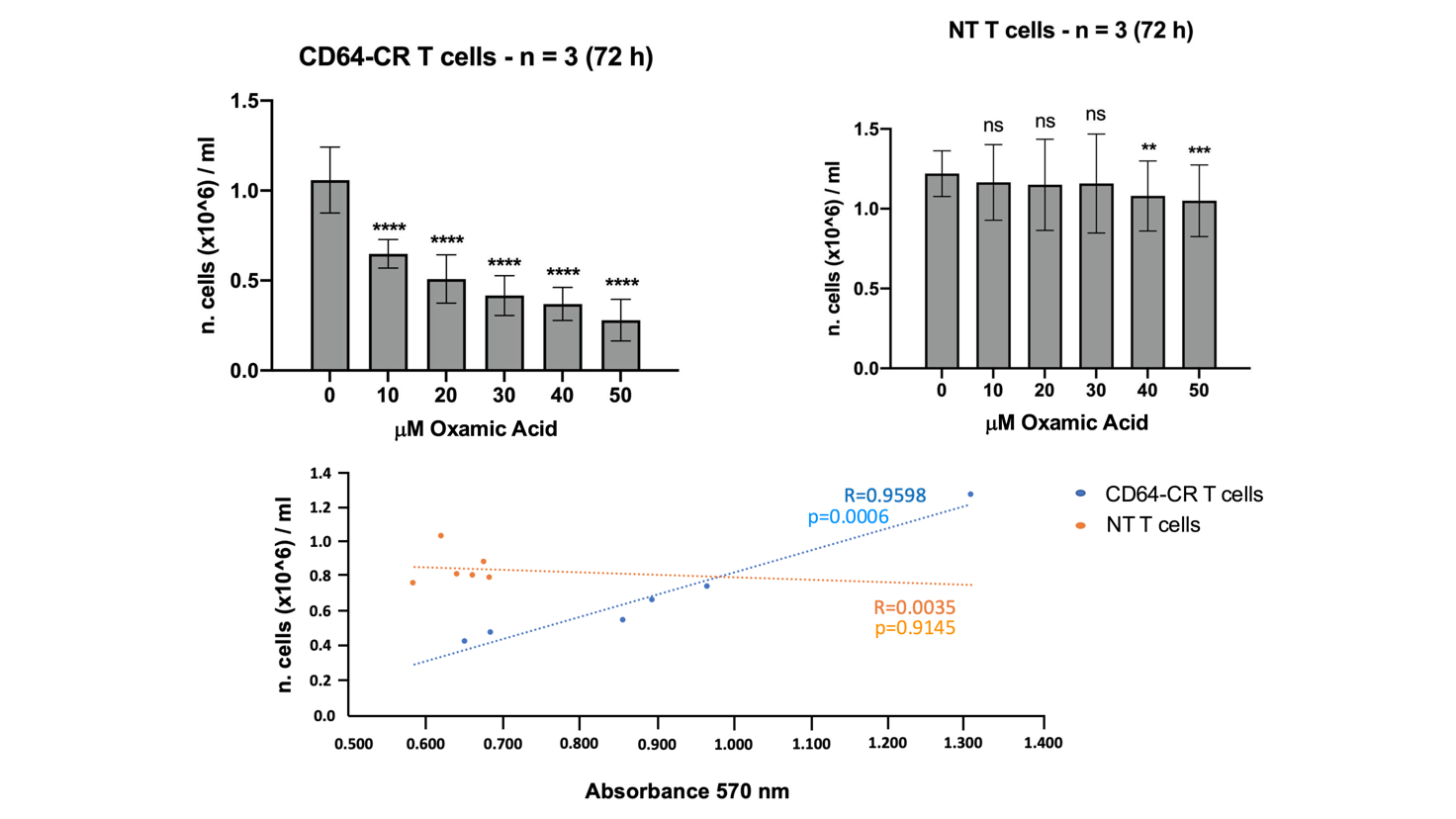


**Figure 3 S. Oxamic Acid preferential inhibition of CD64-CR T cell proliferation**

Upper Panel. CD64-CR T cells and NT T cells were thawed, washed twice, and resuspended at a concentration of 1 x 10^5^ cells per ml in RPMI-1640 complete medium, which was supplemented with 10 ng/ml IL-7 and 5 ng/ml IL-15. This was done in the presence or absence of the indicated concentrations of oxamic acid. After a 72-h, incubation in 96-well plates, at 37°C in 5% CO_2_, the cells were stained with trypan blue and counted. Generally, at least 95% of the cells were found to be trypan blue negative. The means ± SEM (Standard Error of the Mean) are indicated. Statistical analysis was performed using two-way ANOVA, with **p<0.01, and ***p<0.001, ****p<0.0001 indicating statistical significance. Lower Panel. A Spearman Rank correlation analysis was conducted, revealing that the CD64-CR T cell total number correlated with the absorbance at 570 nm from the MTT assay. The coefficients R of CD64-CR T cells and NT T cells are indicated while the p values were 0.0006 and 0.9145, respectively. The figure shows one representative experiment out of three, all yielding similar results.

**
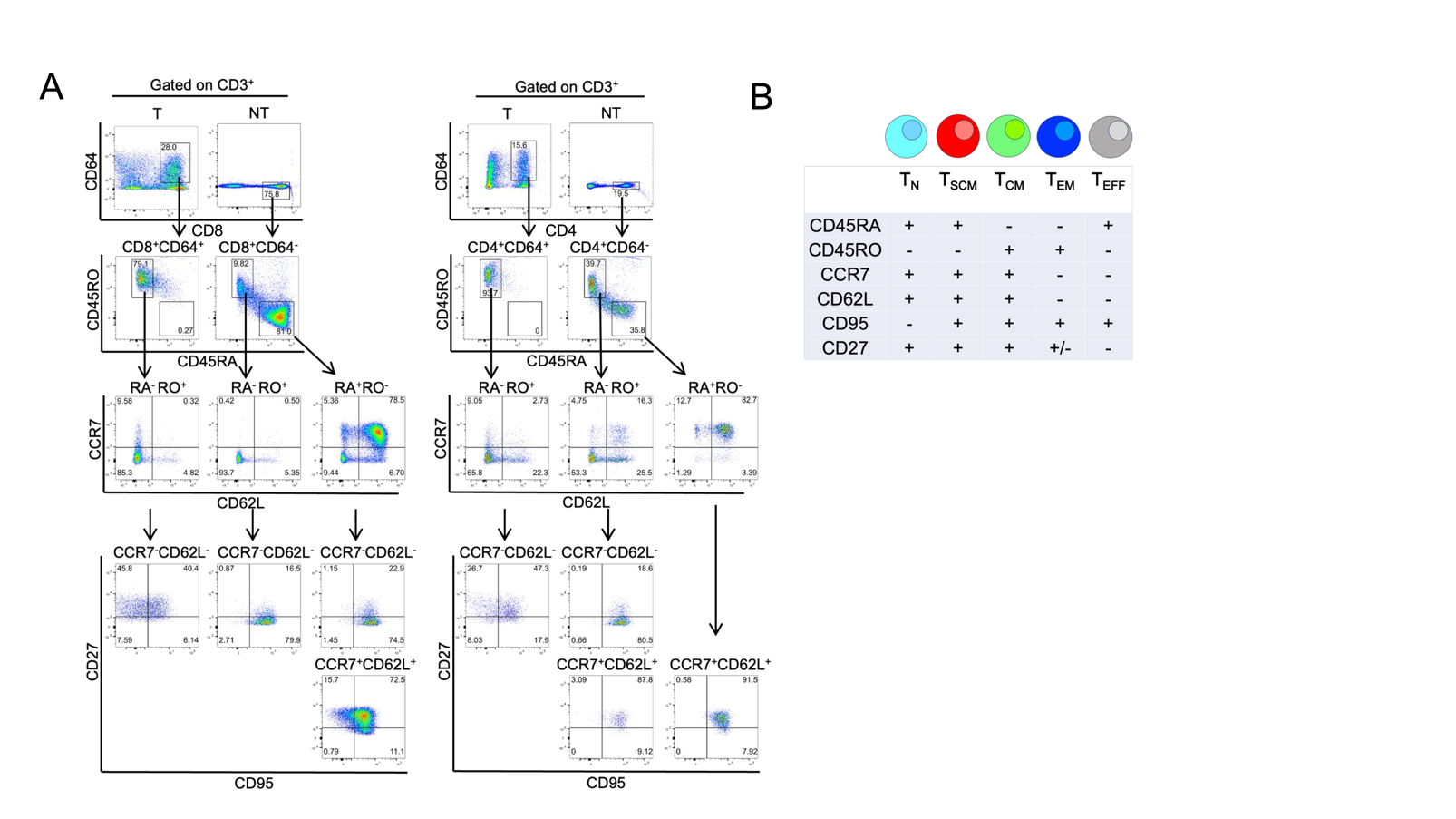
**

**Figure 4 S. Gating strategy of flow cytometry analysis of CD64-CR T cells at 22 days post-transduction.** (**A**) Dot plots are shown as a representative example of the gating strategy used for identifying the frequency of stem cell memory (scm) T cells, central memory (cm) T cells, (em) effector memory T cells, and effector (eff) T cells in the CD64+CD3+ cells. (**B**) Panel of T cell differentiation markers used for identifying different subsets of T cells. At least 50000 events were collected during sample acquisition.

**Figure 5 S.** The animation illustrates CD64-CR polarization in CD64-CR T cells interacting with HT-29 CRC cells at the contact interface.

**
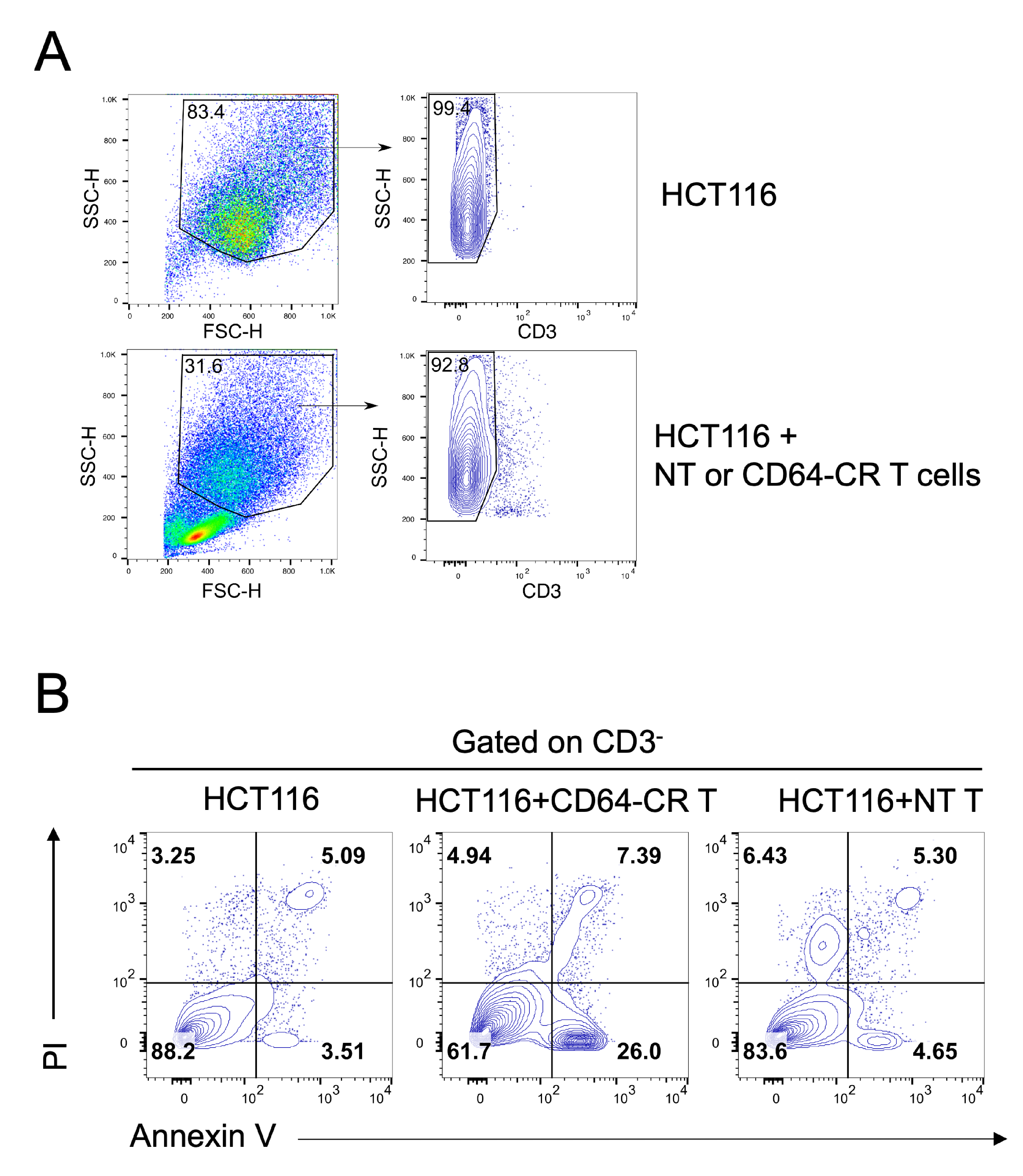
**

**Figure 6 S.** **Flow cytometry cytotoxicity assay confirms CD64-CR as a cytotoxic triggering molecule in engineered T cells**.

(**A**) Left, dot plot analysis of HCT116 and CD64-CR T cell physical parameters shows distinct SSC-H. Right panels show cells gated on SSC-High and CD3 negative. (**B**) shows the flow cytometry analysis of HCT116 cell viability after incubation with CD64-CR T or NT T cells (E:T 2:1). Following overnight coculture, the cells were stained with an APC-conjugated anti-human CD3 mAb, FITC-Annexin V, and propidium iodide (PI) solution and analyzed by flow cytometry. HCT116 cells were identified by posting an electronic gate on CD3^-^ cells. Contour plots are representative of four independent experiments. At least forty thousand cell events were collected. The percentages of cells are indicated in the quadrants. To be noted: this Figure may contain the following caveats: 1) flow cytometric cytotoxicity assays only capture a moment in time. As such, target cells that are killed and disintegrate before or during sample collection are not accounted and 2) anti-CD3 antibodies can cause internalization of the TCR that could lead to an overestimated target cell killing.
